# Supplementary material for: CD47-amyloid-β-CD74 signaling triggers adaptive immunosuppression in sepsis
Source: EMBO Rep. 2025 Apr 4;26(10):2683–714. doi: 10.1038/s44319-025-00442-4 (PMC12116991; doi:10.1038/s44319-025-00442-4)
Supplement: Supplementary file 1 — Appendix [file 44319_2025_442_MOESM1_ESM.pdf]

Appendix

Appendix Figure S1 2
Appendix Figure S2 3
Appendix Figure S3 4
Appendix Figure S4 5
Appendix Figure S5 6
Appendix Figure S6 9
Appendix Figure S7 10
Appendix Figure S8 11
Appendix Figure S9 12
Appendix Figure S10 13
Appendix Figure S11 14
Appendix Figure S12 15
Appendix Figure S13 16
Appendix Figure S14 17
Appendix Figure S15 17
Appendix Figure S16 18
Appendix Figure S17 19
Appendix Figure S18 20
Appendix Figure S19 21
Appendix Figure S20 21
Appendix Figure S21 22
Appendix Figure S22 23
Appendix Table S1 24

Appendix Figure S1

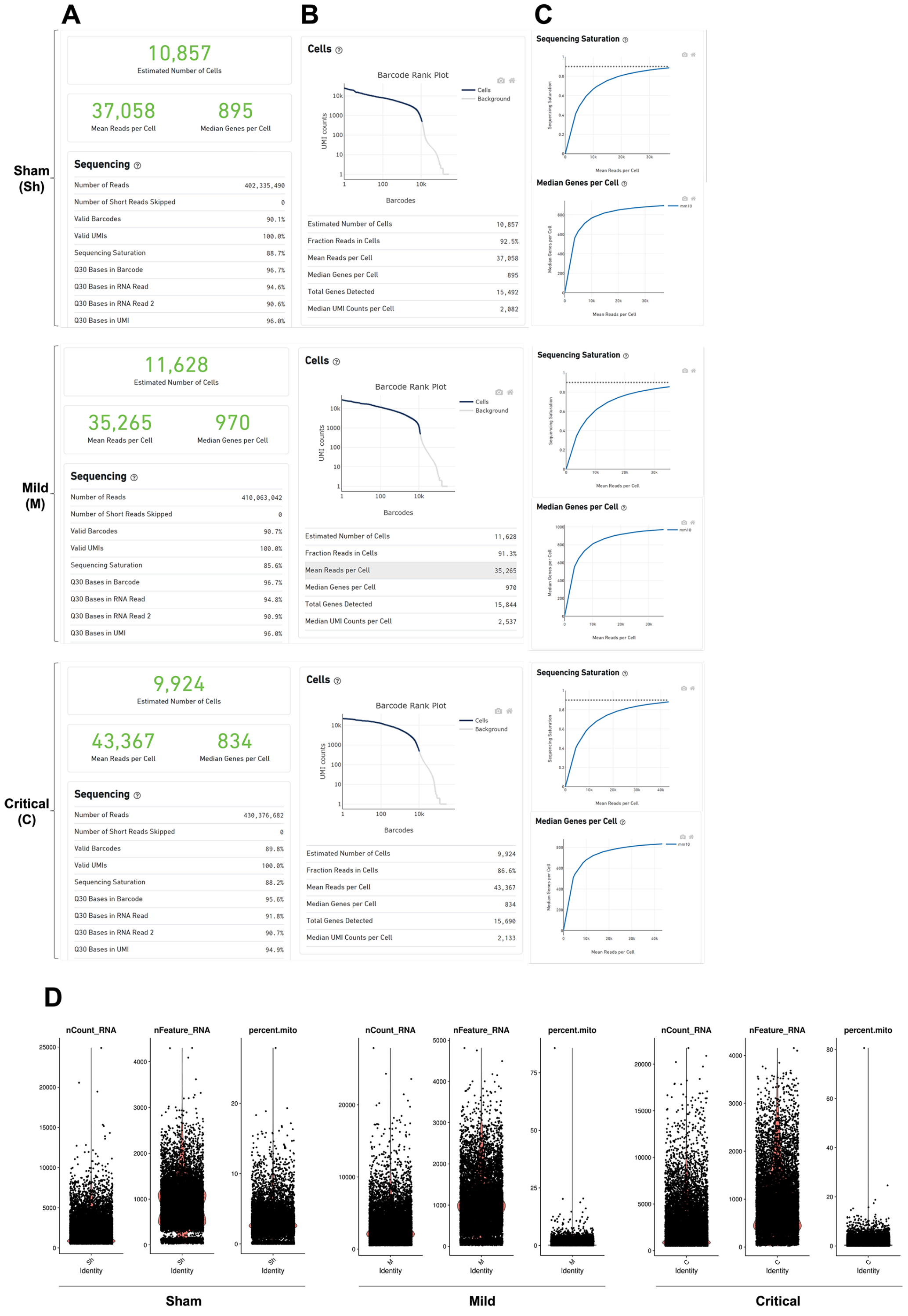

Appendix Figure S1. Quality control of scRNA-seq. (A) The basic information of the scRNA-seq. 10857 PBMCs were sequenced at a depth of 37058 reads/cell in the sham surgery group, 11628 PBMCs were sequenced at a depth of 35265 reads/cell in the mild infection group, and 9924 PBMCs were sequenced at a depth of 43367 reads/cell in the critical infection (sepsis)

group. The median number of genes detected per cell in each group was 895, 970, and 834, and the sequencing saturation rates per cell were 88.7%, 85.6%, and 88.2%, respectively. (**B**) The detailed parameters showing the estimated number of cells, the mean reads per cell, the median genes per cell, and the quality control parameters. (**C**) The graphs showing the sequencing saturation and the median gene per cell. Different degrees of infection did not yield any notable bias in RNA quality. **(D)** Seurat analysis of the scRNA-seq transcriptomic data from the healthy control (Sham), mild infection (Mild), and critical infection (Critical) group. The left column shows the number of unique RNA molecules detected in each cells (nCount-RNA). The middle column shows the median value of feature RNA. The right panel shows the percentage of reads mapping to the mitochondrial genome (percent.mt). The mitochondrial gene expression higher than 25% were filtered out.

Appendix Figure S2

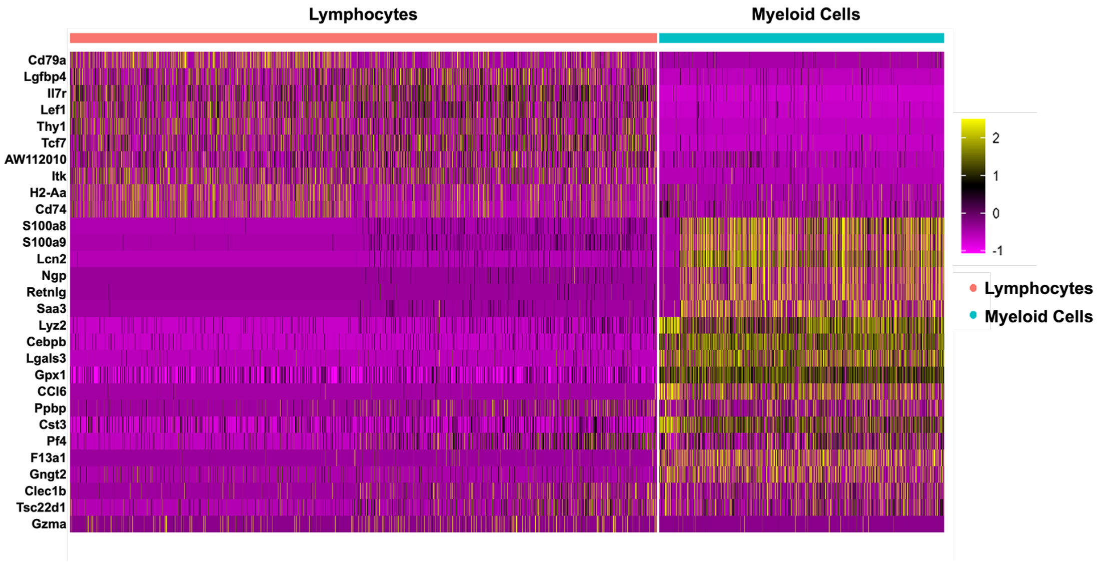

Appendix Figure S2. Expression heatmap showing that the PBMCs were classified into two major populations: lymphocytes and myeloid cells.

Appendix Figure S3

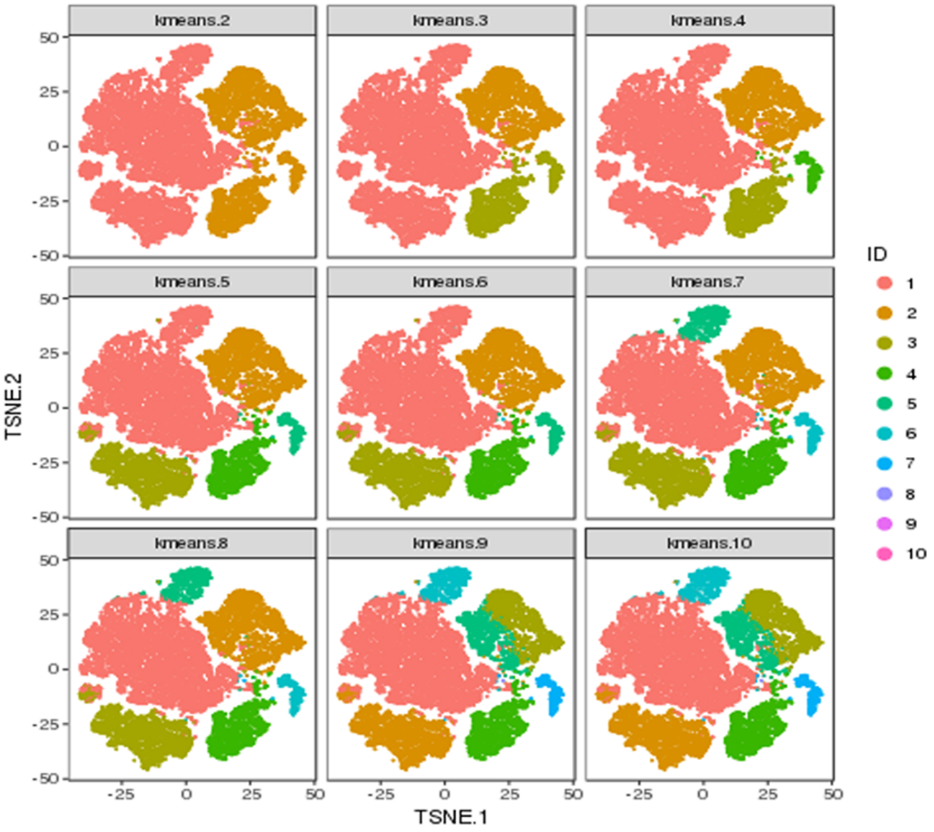

Appendix Figure S3. Applying k-means clustering. The t-SNE analysis applies k-means clustering with k = 9 on the CyTOF data, resulting in a clear distinction between 10 clusters.

Appendix Figure S4

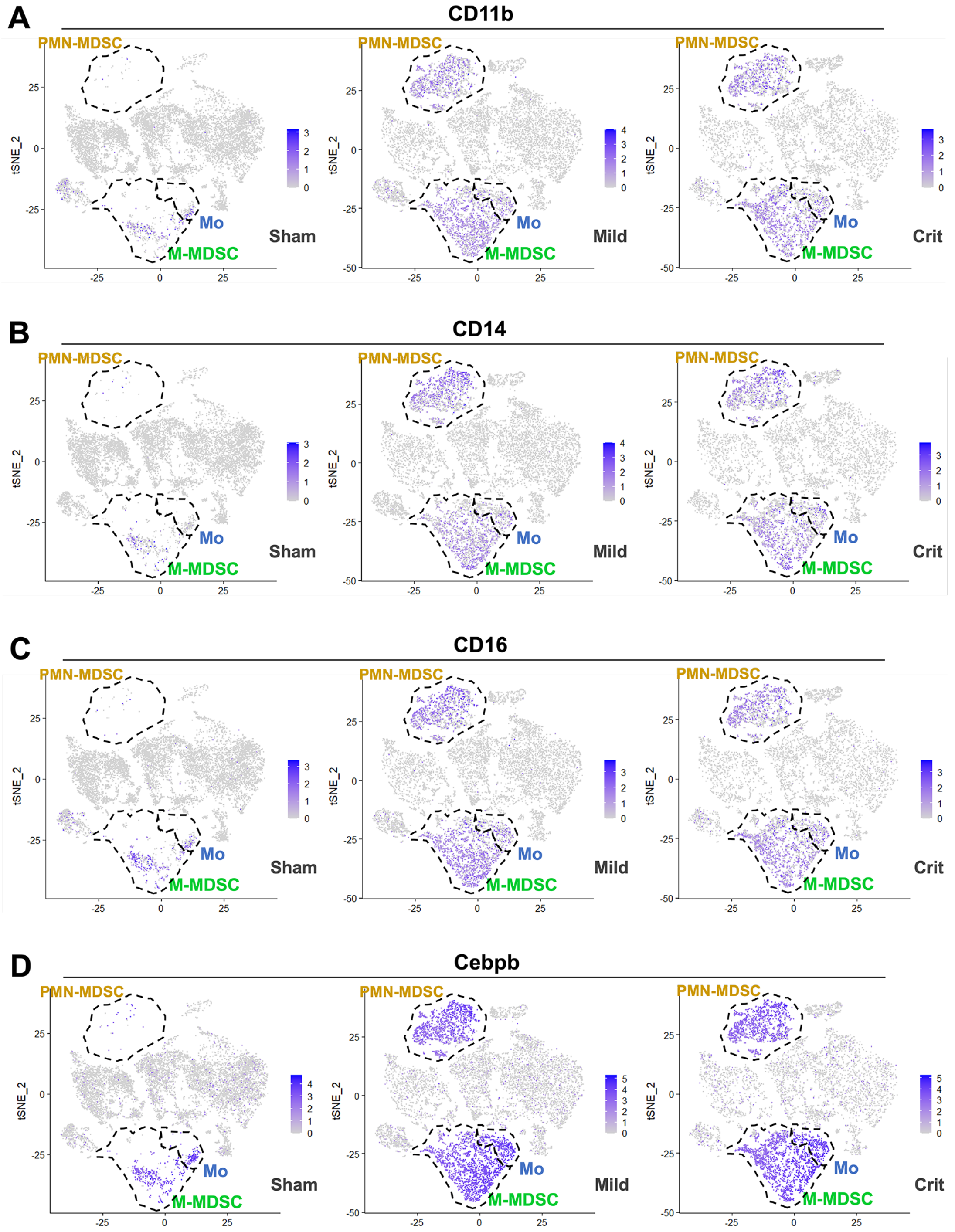

Appendix Figure S4. Common markers shared by all myeloid cells. The t-SNE analysis showing the common markers shared by all myeloid cells (including PMN-MDSCs, M-MDSCs, Mo; these cells were circled by dash line): (**A**) CD11b, (**B**) CD14, (**C**) CD16, (**D**) Cebpb.

Appendix Figure S5

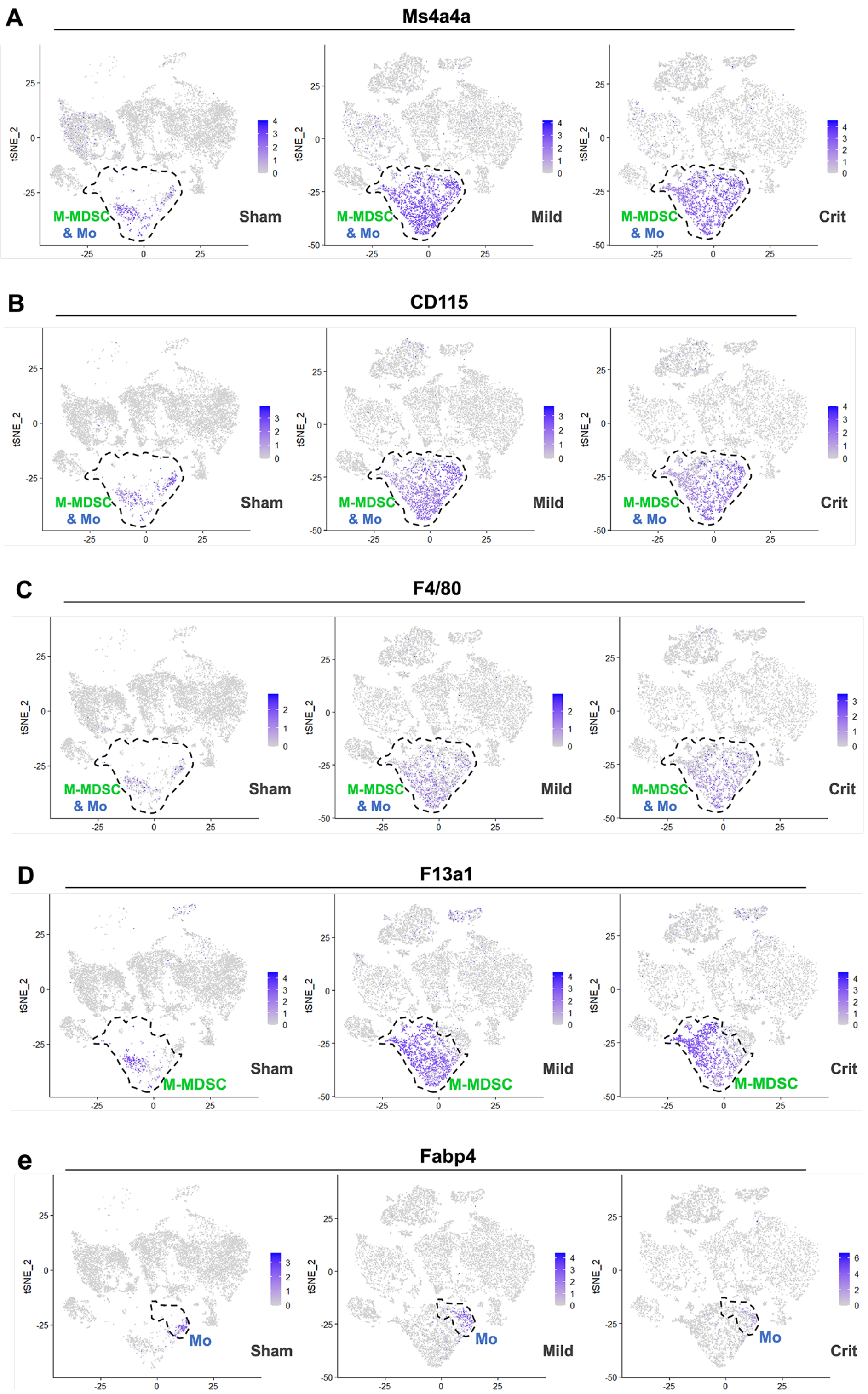

Appendix Figure S5. Markers to identify M-MDSCs and Mos/Mφs. The t-SNE analysis showing the markers shared by M-MDSCs and Mo, including (**A**) Ms4a4a, (**B**) CD115, and (**C**) F4/80. The special marker of M-MDSCs is (**D**) F13a, and the special marker of Mo is (**E**) Fabp4. The molecular markers used to distinguish M-MDSCs from Mos/Mφs were F13a1 and

Fabp4(Chang *et al.*, 2021; Ding *et al.*, 2019; Liang *et al.*, 2019; Porrello *et al.*, 2018; Steen *et al.*, 2017; Xiao *et al.*, 2021). F13a1 is the gene encoding Factor XIII. Recent studies have reported that macrophages expressing F13a1 exhibit an immunosuppressive phenotype(Ding *et al.*, 2019; Porrello *et al.*, 2018). F13a1 was specifically expressed in M-MDSCs, and Fabp4 was used to identify Mos/Mφs, according to published studies(Chang *et al.*, 2021; Liang *et al.*, 2019; Steen *et al.*, 2017; Xiao *et al.*, 2021).

Appendix Figure S6

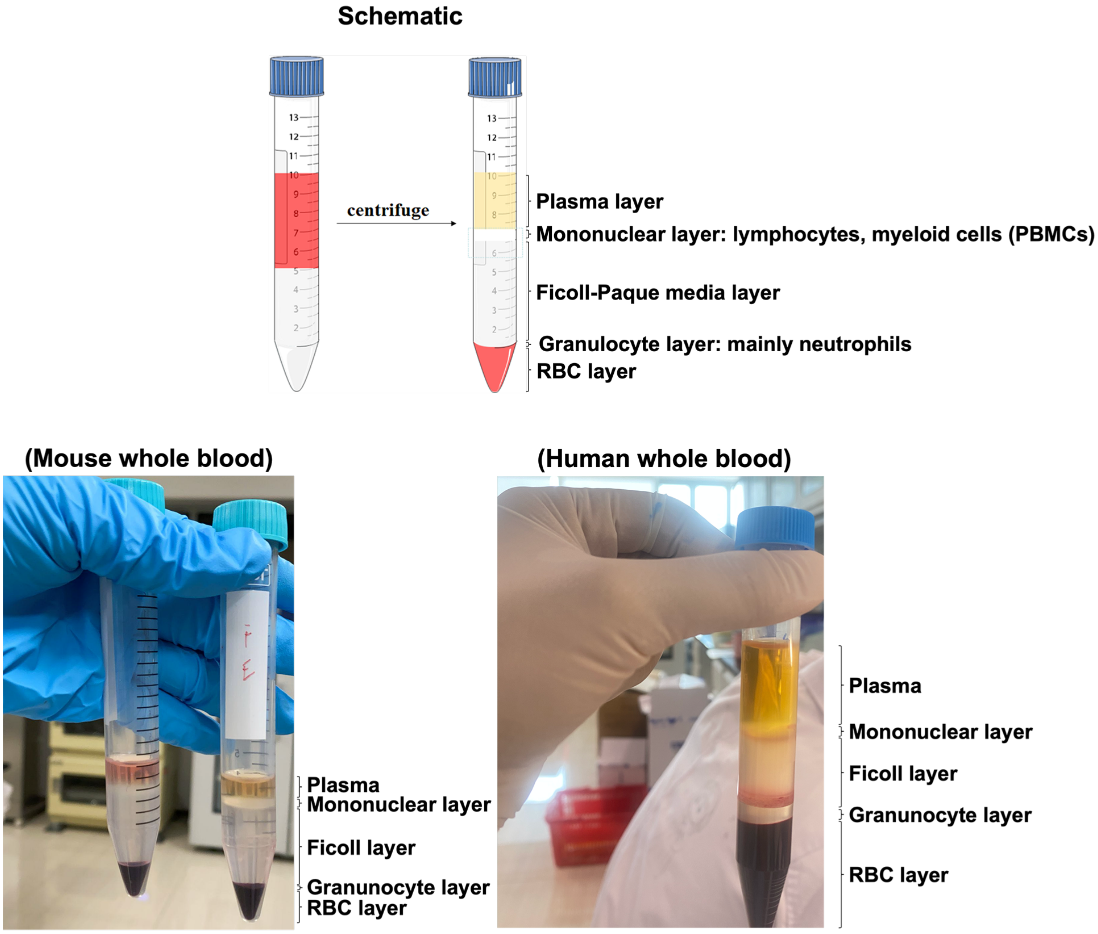

Appendix Figure S6. Standard Ficoll gradient centrifugation to isolate PBMCs from peripheral blood. After centrifugation, the upper layer of plasma, the lower layer of Ficoll-Paque media, the Granulocytes layer, and RBC layer were discarded. The buffy coat that contains all mononuclear cells (including lymphocytes and myeloid cells) was aspirated. There is a thick Ficoll-Paque media layer between the granulocytes and the buffy coat, so the neutrophils can be removed cleanly. The experimental procedure was added into the *Methods* section

Appendix Figure S7

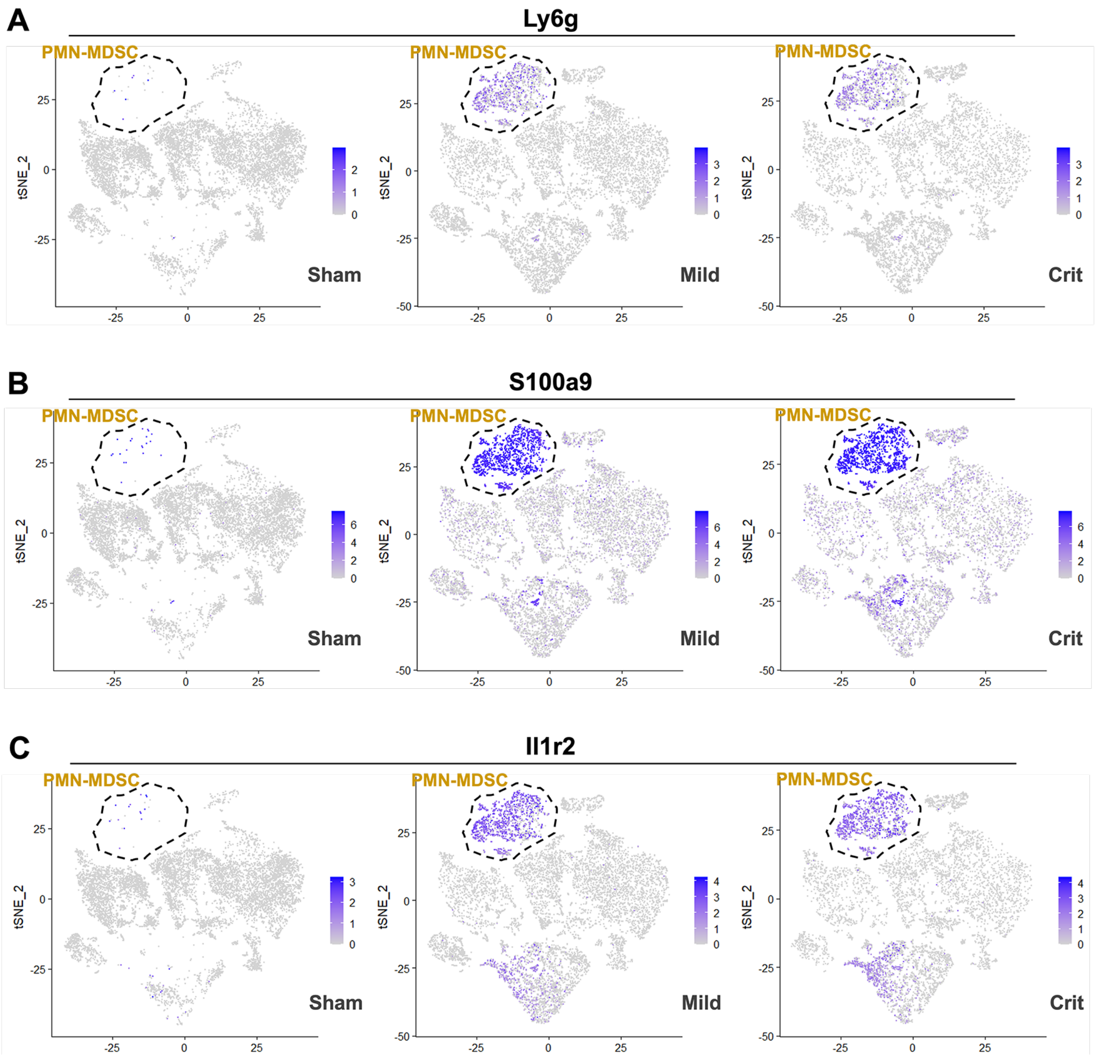

Appendix Figure S7. Markers to identify PMN-MDSCs. The t-SNE analysis showing the markers of PMN-MDSCs, including Ly6g (**A**), S100a9 (**B**), Il1r2 (**C**).

Appendix Figure S8

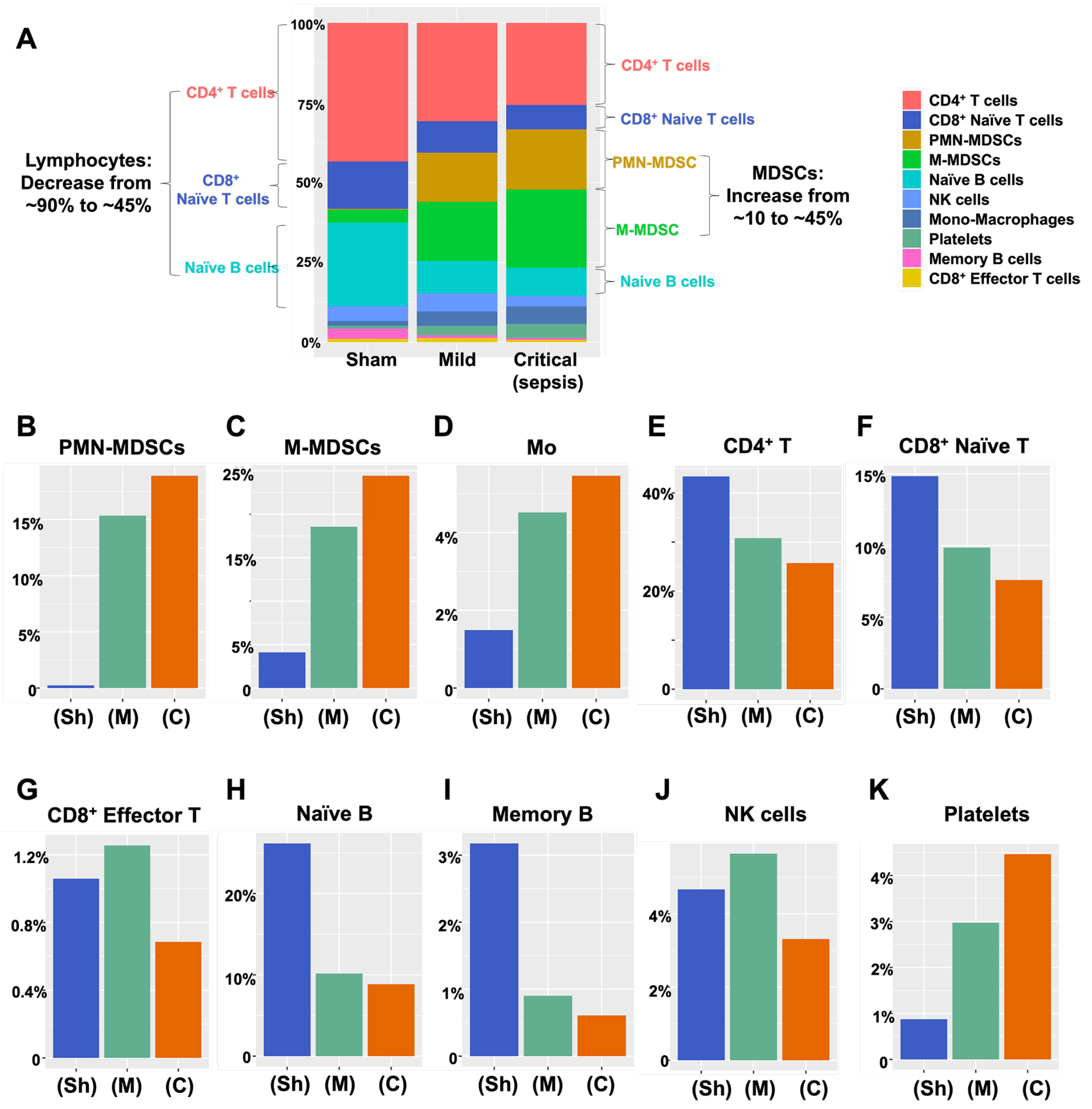

Appendix Figure S8. The trend of change of each cell cluster. (A) Histogram showing the trends for changes in the ratio of each cell population in PBMCs as the severity of infection worsened. **(B-K)** The change in the proportion of PMN-MDSCs **(B)**, M-MDSCs **(C)**, Mo **(D)**, CD4$^+$ T cells **(E)**, CD8$^+$ naïve T cells **(F)**, CD8$^+$ effector T cells **(G)**, Naïve B cells **(H)**, Memory B cells **(I)**, NK cells **(J)**, and platelets **(K)**.

Appendix Figure S9

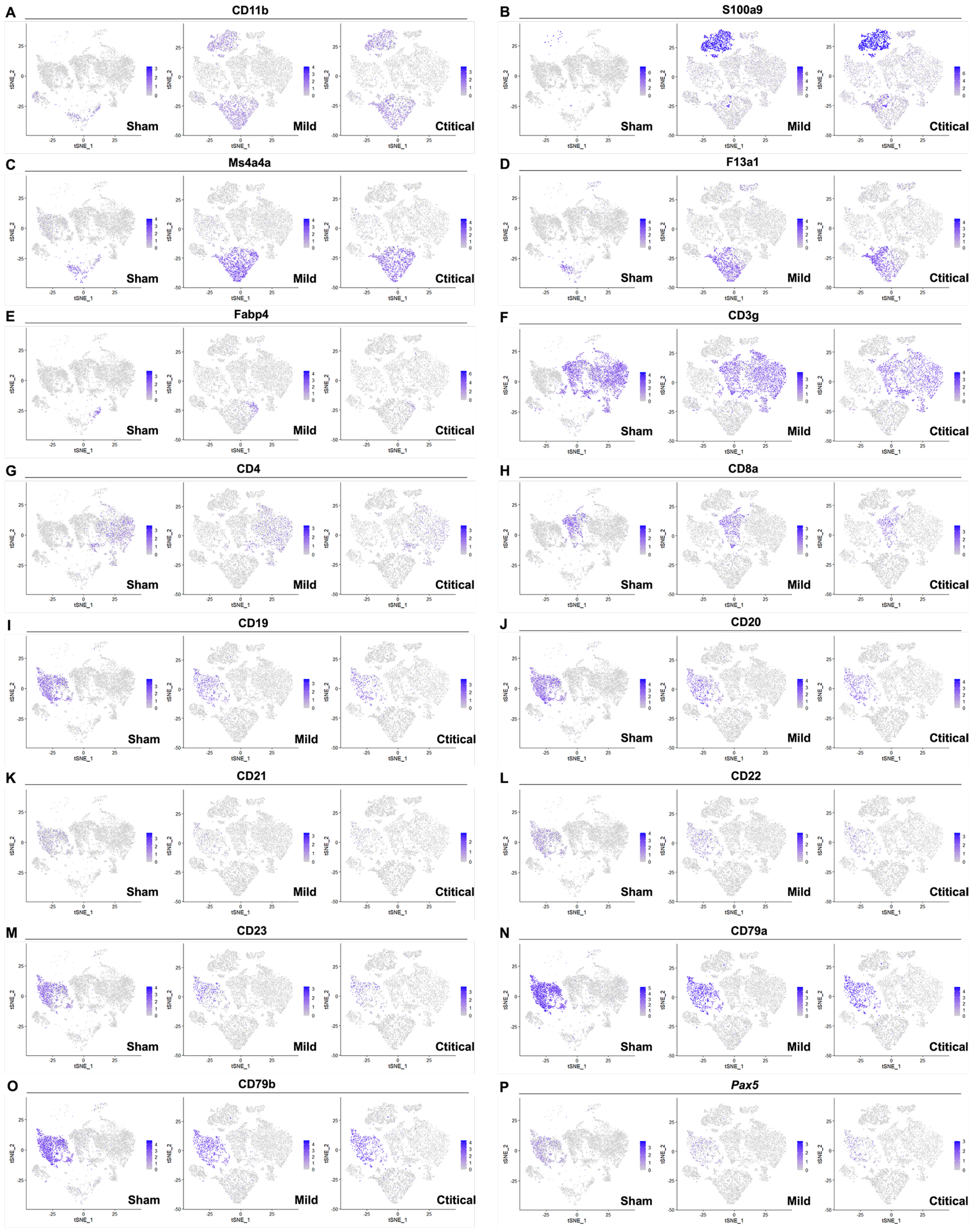

Appendix Figure S9. The expression level of the marker genes for MDSCs and T/B cells. The t-SNE plots showing the expression of CD11b (**A**), S100a9 (**B**), Ms4a4a (**C**), F13a1 (**D**), Fabp4 (**E**), CD3g (**F**), CD4 (**G**), CD8a (**H**), CD19 (**I**), CD20 (**J**), CD21 (**K**), CD22 (**L**), CD23 (**M**), CD79a (**N**), CD79b (**O**), *Pax5* (**P**).

Appendix Figure S10

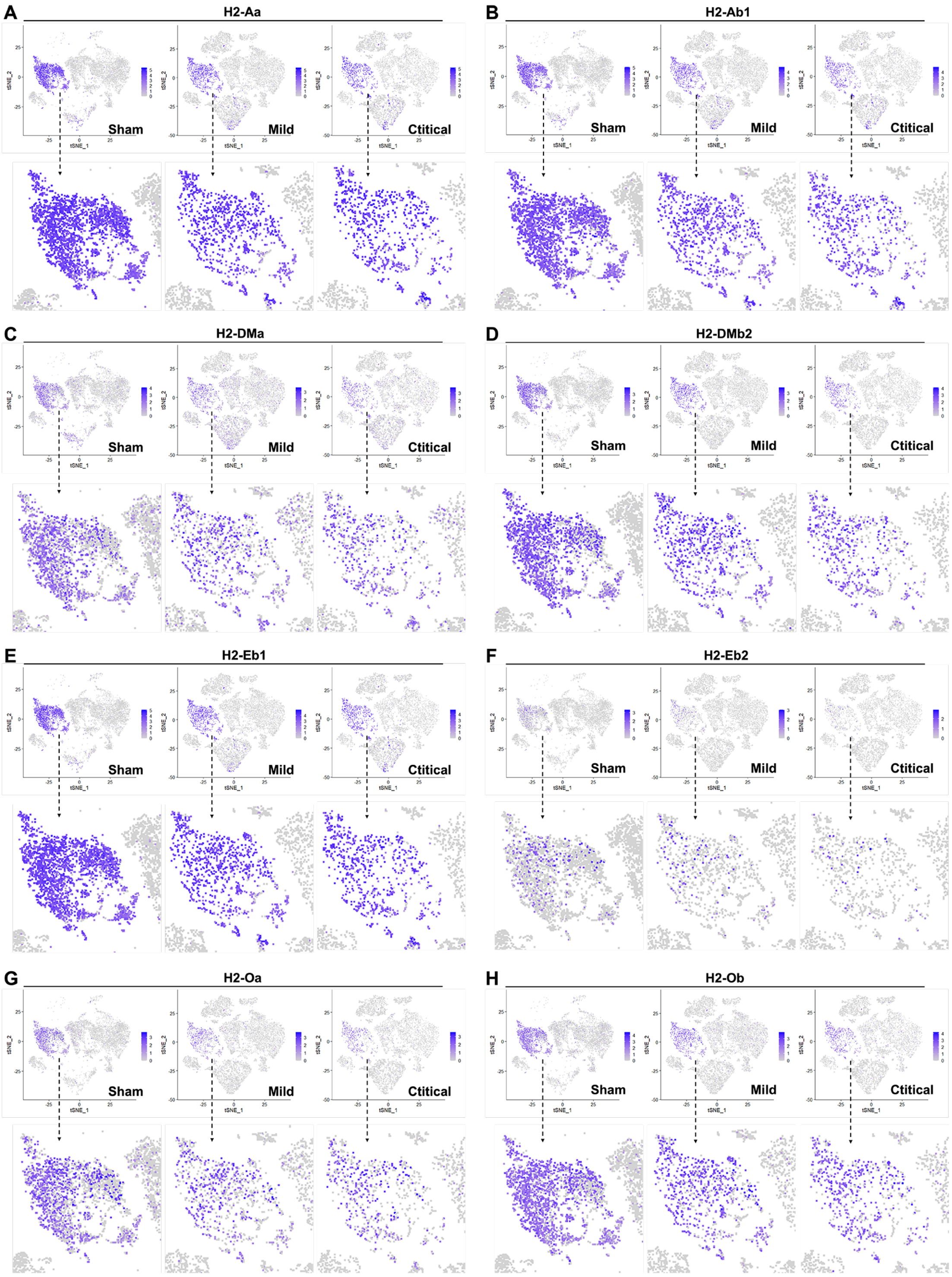

Appendix Figure S10. There was a significant downregulation of MHC class II genes when sepsis occurs. The t-SNE plots showing the expression of (**A**) H2-Aa (corresponding to HLA-DQA), (**B**) H2-Ab1 (HLA-DQB1), (**C**) H2-DMa (HLA-DMA), (**D**) H2-DMb2 (HLA-DMB2), (**E**) H2-Eb1 (HLA-DR1), (**F**) H2-Eb2 (HLA-DR2), (**G**) H2-Oa (HLA-DOA), (**H**) H2-Ob (HLA-DOB).

Appendix Figure S11

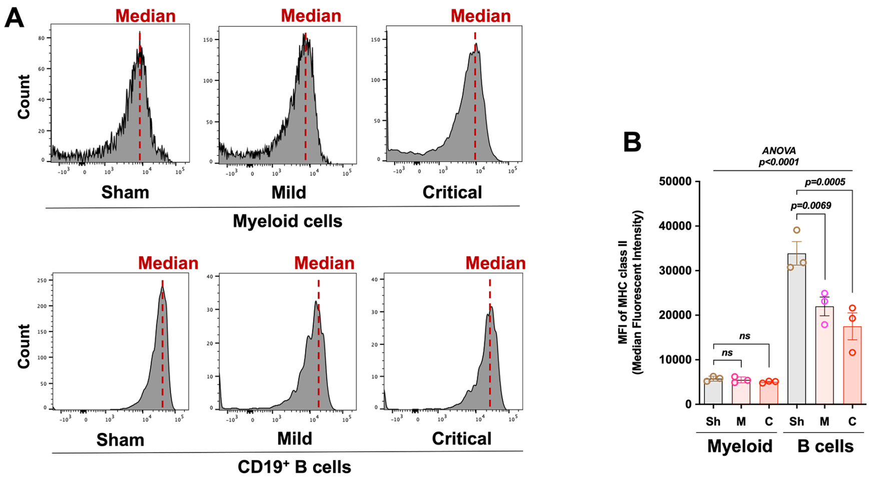

Appendix Figure S11. The trend of change of each cell cluster. (A) The median fluorescence intensity (MFI) was calculated to evaluate the expression level of MHC class II molecules on myeloid cells and B cells. **(B)** MFI quantification and significance were assessed by *one-way ANOVA* followed by *Tukey's multiple comparison test*, n=3

Appendix Figure S12

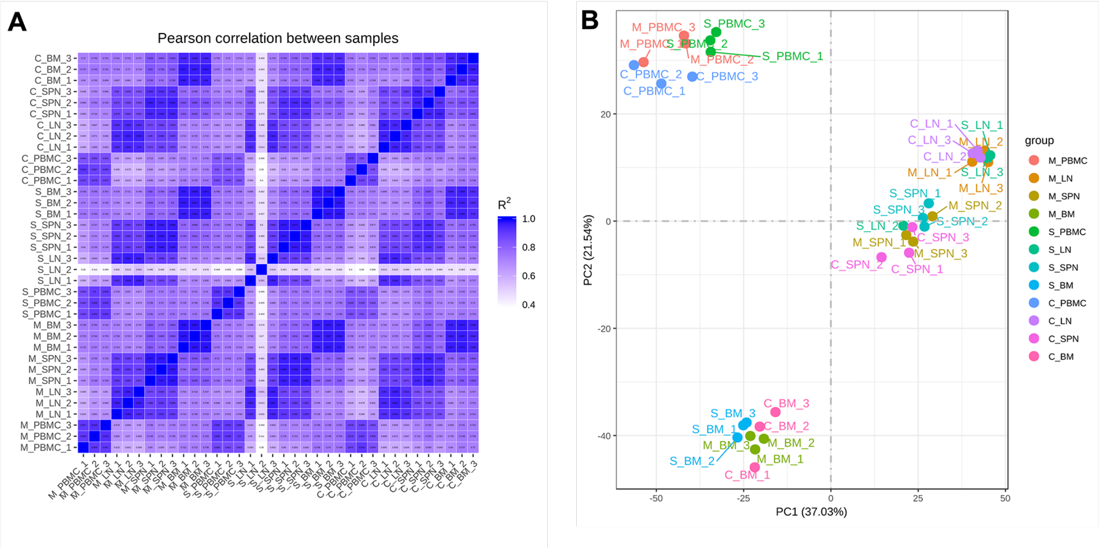

Appendix Figure S12. Quality control of the RNA-seq. Three parallel samples from each group were analyzed, and the intra-group and inter-group correlation coefficients for samples were calculated. **(A)** The square of the Pearson correlation coefficient (R2) in each group indicated good reliability of the experiments and high similarity among the sample expression patterns. **(B)** The principal component analysis (PCA) showed that the samples from different groups were scattered, while the samples from the same group were clustered, indicating good replication within each group and significant differences among the groups.

Appendix Figure S13

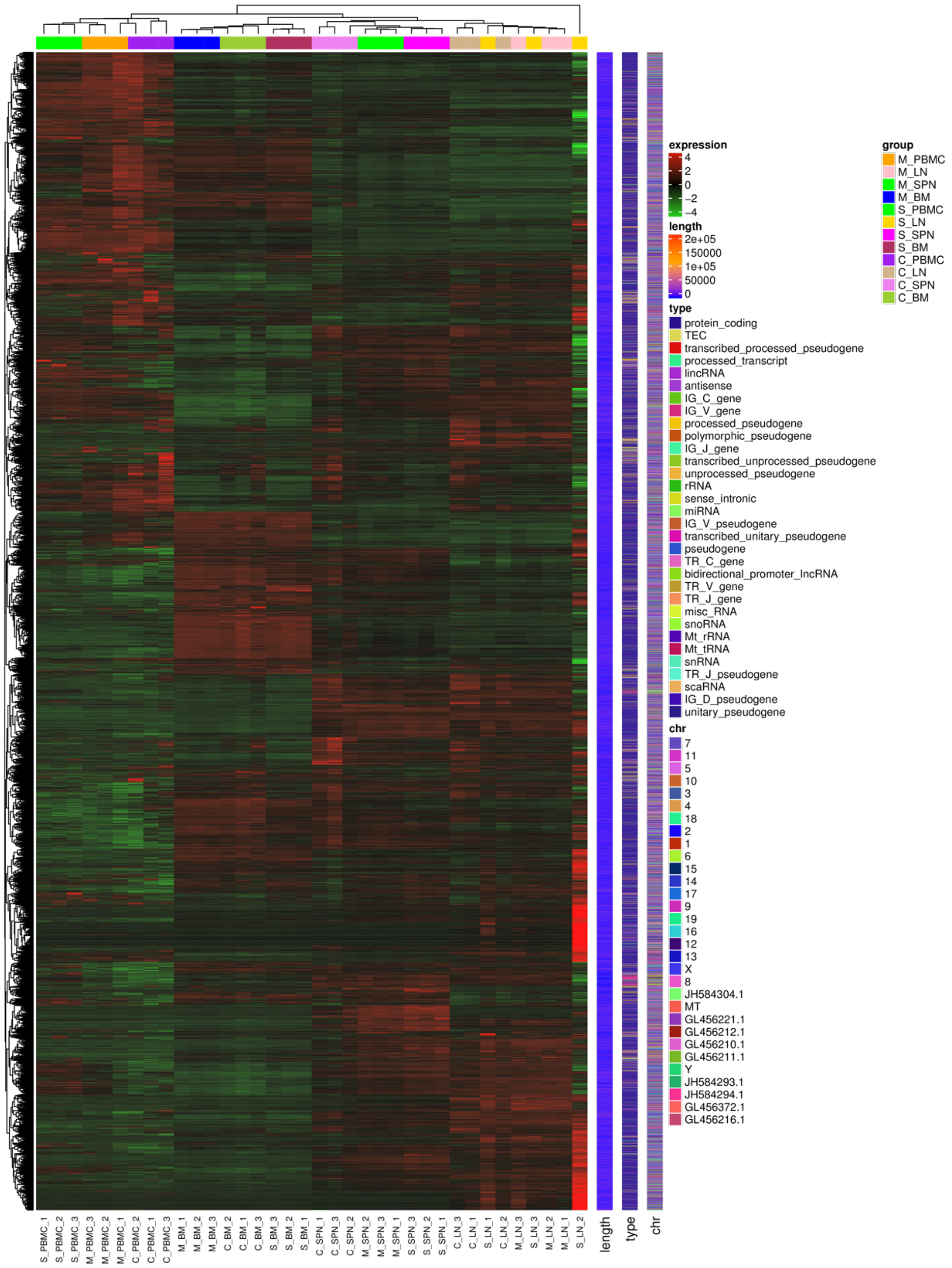

Appendix Figure S13. The heatmap of the RNA-seq.

Appendix Figure S14

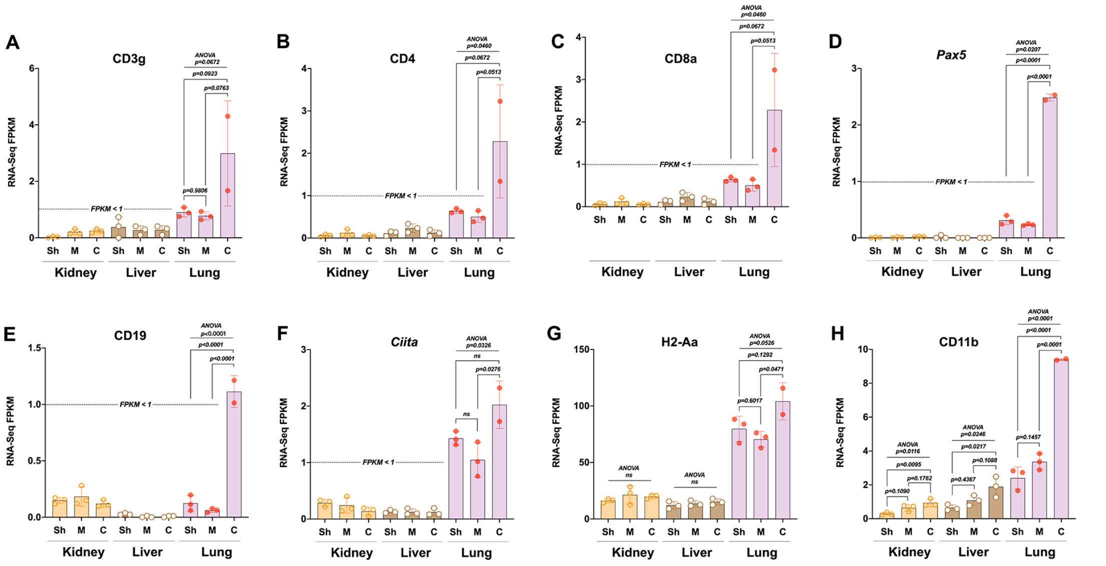

Appendix Figure S14. Bulk-RNA-seq of the major organs. Samples from major organs, including the kidney, liver and lung, were collected 8 hours after the sham or CLP surgery and analyzed by RNA-seq (3 parallel samples per group). *One-way ANOVA* followed by *Tukey's multiple comparison test* were used to compare gene expression in a certain organ.

Appendix Figure S15

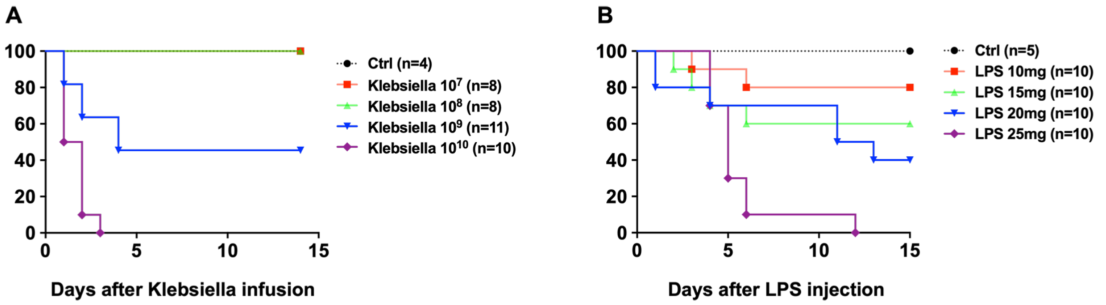

Appendix Figure S15. Survival of Klebsiella pneumonia-induced sepsis model. (A) The pneumonia-induced sepsis model was established by injecting *Klebsiella pneumoniae* directly into the lungs of mice via tracheal intubation. Four different doses of 10^{7} CFU, 10^{8} CFU, 10^{9} CFU and 10^{10} CFU were used to evaluate the survival rate. When doses of 10^{7} and 10^{8} CFU were given, none of the mice died, while 10^{10} CFU killed all the mice within 3 days. Infusion of 10^{9} CFU resulted in the death of approximately 45% of the mice, and this dose was chosen for

subsequent experiment. **(B)** The endotoxin model was established by i.p. injection of different doses of LPS (10/15/20/25 mg/kg) to evaluate the survival rate.

Appendix Figure S16

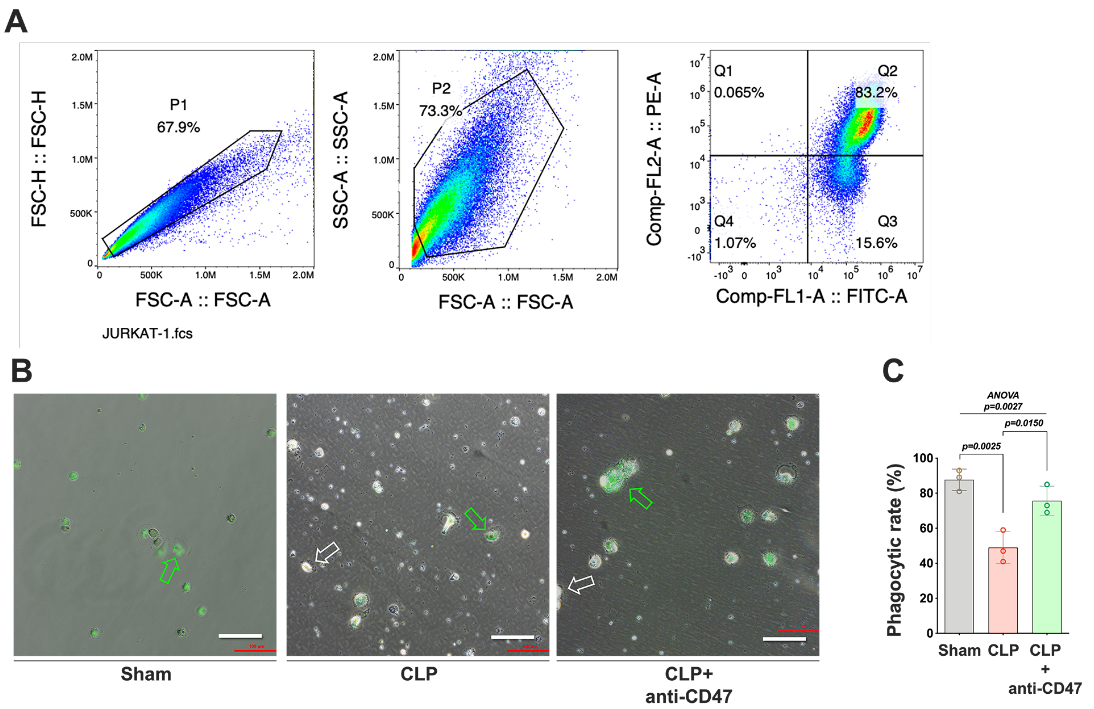

Appendix Figure S16. The CD47-SIRPα signaling reduces the efferocytotic ability of myeloid cells. (A) Jurkat cell was used as a model cell to test the effect of CD47 on phagocytosis of apoptotic T cells. 1×10^6 Jurkat cells were treated with *Staurosporine* (50uM at 37°C) for 2 hours to induce apoptosis. The ratio of apoptotic cells was assessed by flow cytometry using the Annexin V-FITC/PI Apoptosis Detection Kit. Under this treatment condition, > 80% of Jurkat cells were apoptotic. **(B)** The phagocytosis rates were calculated by using GFP-labeled apoptotic cells (green arrows indicate the myeloid cells that were phagocytizing apoptotic cells, and white arrows indicate non-phagocytizing myeloid cells). Scale bars: 100 μm. **(C)** Phagocytics rate (%) was calculated. Data were shown as means ± S.D. (n=3, biological replicates) and were assessed by *one-way ANOVA* followed by *Tukey's multiple comparison test*. The exact *p* values were annotated above the bars.

Appendix Figure S17

A

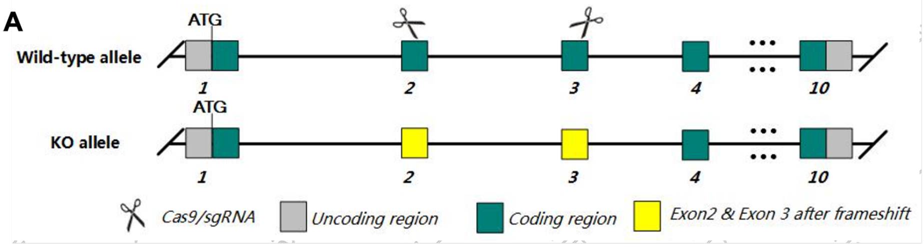

B

gRNA No.	gRNA Sequence（5'→3'）	PAM
5S1	CCCTTGCATCGTCCGTAATG	TGG
3S1	ATCAGCCTGTTCTTACGAGG	AGG

C

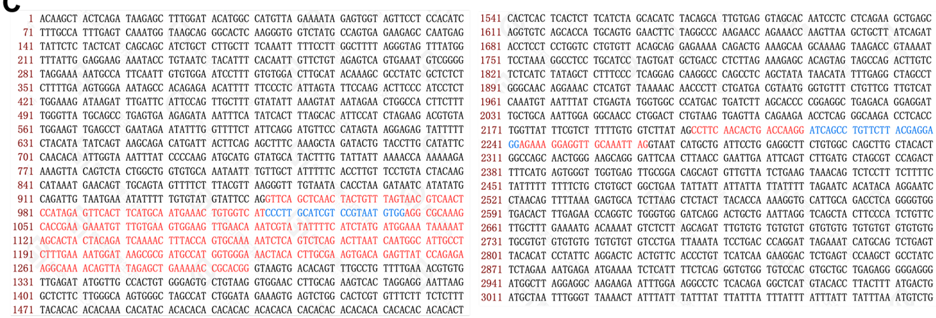

D

aacgtcaactccatagagttcacttcatgcaatgaaact------*1228bp*------ggaggagaaaggaggttgcaaattaggtaatcatgctgatt

E

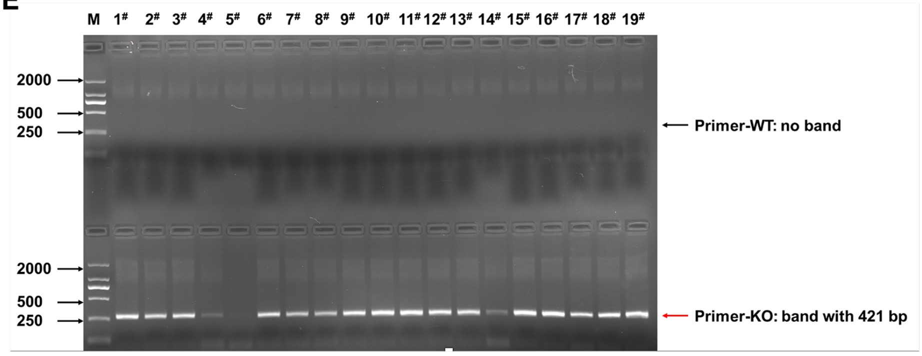

Appendix Figure S17. The generation of CD47-KO mice. (A) The CD47-KO C57BL/6J mice were generated using CRISPR/Cas9 system. In brief, the sgRNAs were designed targeting the specific locations in the 2nd to 3rd exon of murine *Cd47* gene. **(B)** The gRNA sequences. **(C)** The targeted sequence of murine *Cd47* gene (gRNAs are marked in blue, and exon2 and exon3 are marked in red). **(D)** The sequencing results showed that a total of 1228 bases were deleted, accompanied by frameshift mutations. **(E)** Identification of homozygous CD47-KO mice by PCR.

Appendix Figure S18

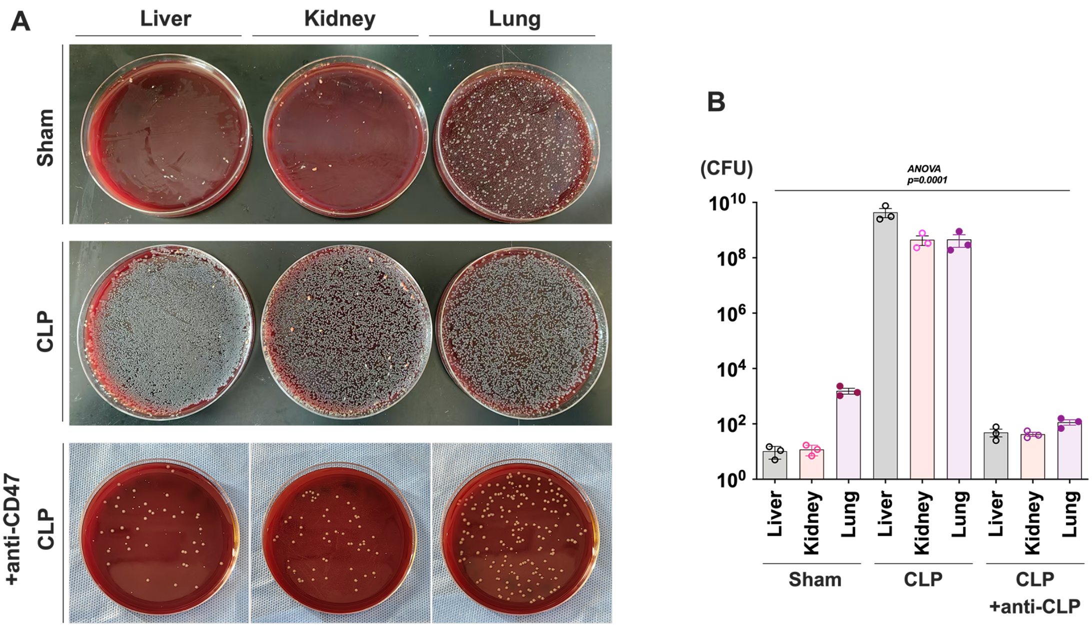

Appendix Figure S18. Blood agar plate culture. (A) Samples of the liver, kidney and lungs were collected from mice that underwent sham, mild CLP or critical CLP surgery. Blood agar plates were used to detect the bacterial load in the tissues. **(B)** The bacterial load (CFUs) in each group was quantified. Data were shown as means ± S.D (n=3, biological replicates) and were assessed by *one-way ANOVA* followed by *Tukey's multiple comparison test*. The *p* value was annotated above the bars.

Appendix Figure S19

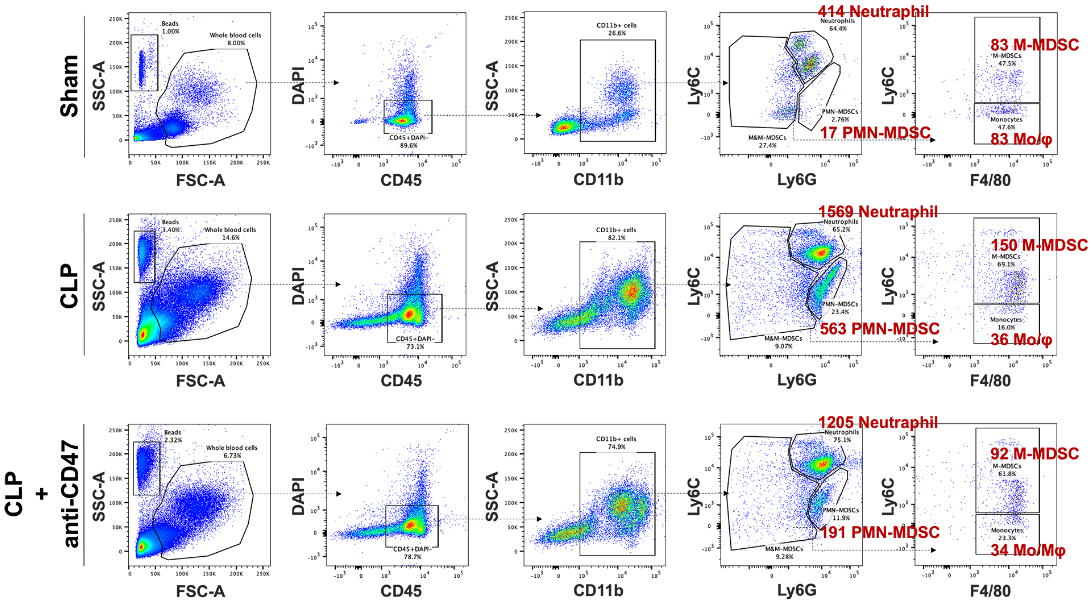

Appendix Figure S19. Flow cytometry data showing the trends of changes in leukocytes. Ly6G^{high}Ly6C^{high} neutrophils, Ly6G^{high}Ly6C^{medium} PMN-MDSCs, Ly6C^{+}F4/80^{+} M-MDSCs and Ly6C^{-}F4/80^{+} monocytes were treated with the anti-CD47 antibody. The absolute cell number was labeled next to the corresponding cell population.

Appendix Figure S20

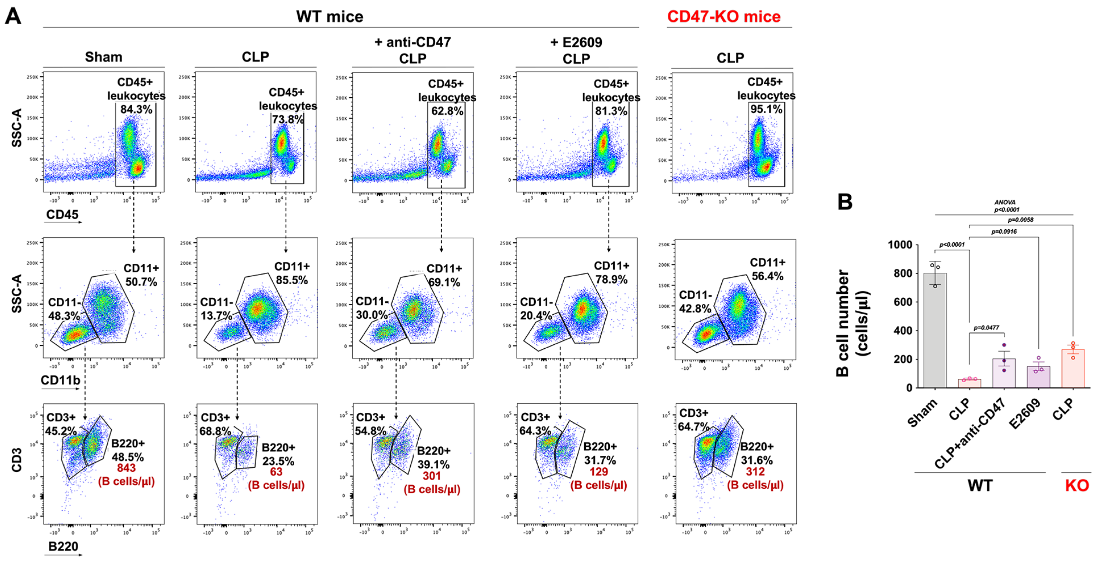

Appendix Figure S20. Flow cytometry data showing that anti-CD47 and E2609 restored B cell numbers. (A) WT mice were treated with the anti-CD47 antibody (2.5 mg/kg, i.v.) or

E2609 (6.5 mg/kg, intraperitoneal) 1 hour after CLP surgery, or CD47-KO mice that underwent CLP surgery. Blood samples were collected from these mice 8 hours after the CLP surgery and analyzed by flow cytometry. **(B)** The absolute number of $B220^{+}$ B cells was calculated using a microbead counter: absolute cell number = positive cell events ÷ bead events × (46800 bead events/50 μl). The simplified formula is (number of cells per tube ÷ number of beads per tube) × 936 (cells/μl). The cells were labeled next to the B-cell population (cells/μl). Data were shown as means ± S.D (n=3, biological replicates) and were assessed by *one-way ANOVA* followed by *Tukey's multiple comparison test*. The exact *p* values were annotated above the bars.

Appendix Figure S21

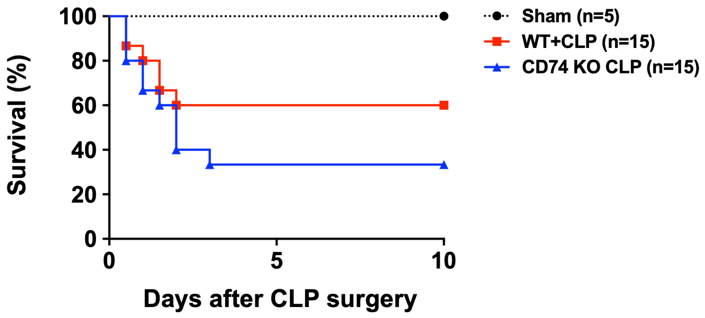

Appendix Figure S21. CD74-KO mice that underwent CLP surgery showed a lower survival rate compared to WT mice. The mice were divided into 3 groups: sham (n=5), WT mice received CLP surgery (n=15), and Cd74-KO mice received CLP surgery (n=15). The survival benefit was evaluated via a *Kaplan–Meier survival analysis*, and significance was assessed by the *log-rank test (p=0.0227).*

Appendix Figure S22

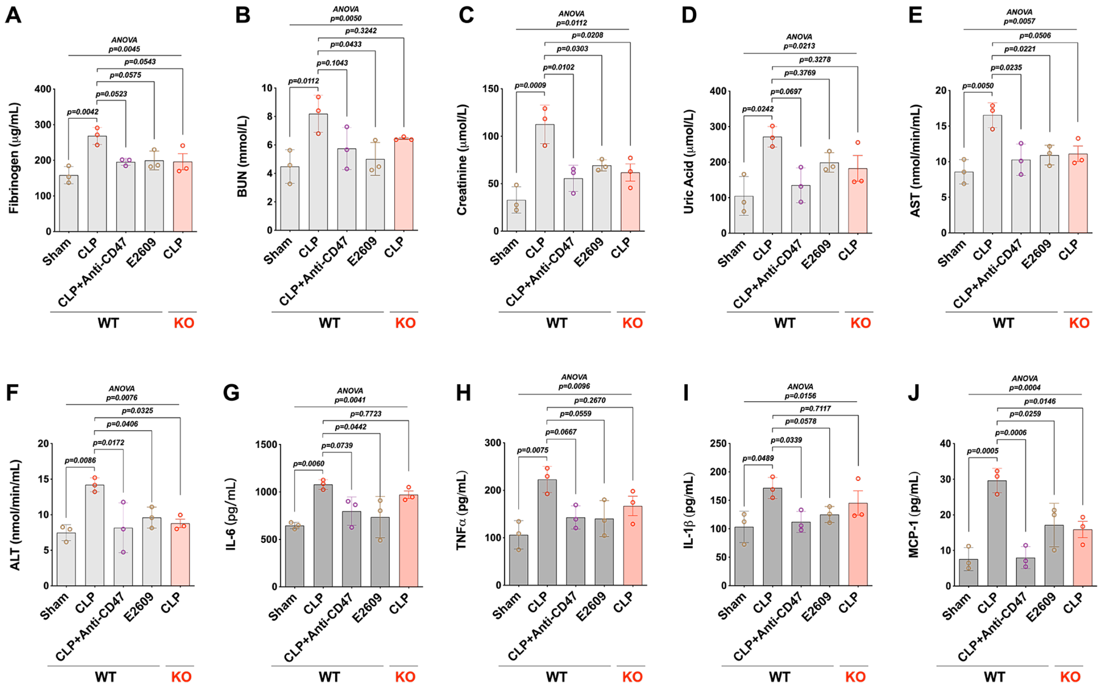

Appendix Figure S22. Blood biochemical tests showed that anti-CD47 and E2609 alleviated liver and kidney damage. (A-F) At 8 h after the CLP surgery, the blood concentrations of fibrinogen, BUN, CREA, UA, AST and ALT were measured. **(G-J)** The concentrations of inflammatory indicators, including IL-6, TNF-α, IL-1β, and MCP, were determined by ELISAs. Data were shown as means ± S.D (n=3, biological replicates) and were assessed by *one-way ANOVA* followed by *Tukey's multiple comparison test*. The exact *p* values were annotated above the bars.

Appendix Table S1

Category	GOID	Description	GeneRatio	BgRatio	pvalue	padj	
BP	GO:0050900	leukocyte migration	104/2368	264/14213	5.22968398977457e-19	2.11062646216345e-15	
BP	GO:0050778	positive regulation of immune response	152/2368	458/14213	1.10877985156426e-18	2.11062646216345e-15	
BP	GO:0031347	regulation of defense response	156/2368	475/14213	1.16523360075273e-18	2.11062646216345e-15	
BP	GO:0098542	defense response to other organism	127/2368	359/14213	2.63636712677447e-18	3.58150474172311e-15	
BP	GO:0050865	regulation of cell activation	151/2368	463/14213	8.77529882993358e-18	7.71474854644572e-15	
BP	GO:0002764	immune response-regulating signaling pathway	104/2368	273/14213	8.85267647916753e-18	7.71474854644572e-15	
BP	GO:0030099	myeloid cell differentiation	122/2368	344/14213	9.93802720373942e-18	7.71474854644572e-15	
BP	GO:0006897	endocytosis	157/2368	492/14213	1.85710304882552e-17	1.26143724591474e-14	E
BP	GO:0002253	activation of immune response	111/2368	308/14213	8.10240745373405e-17	4.89205356706564e-14	
BP	GO:0002757	immune response-activating signal transduction	98/2368	260/14213	1.81291558099872e-16	9.85138326714704e-14	
BP	GO:0002683	negative regulation of immune system process	127/2368	380/14213	4.65949273026716e-16	2.30178940875198e-13	
BP	GO:0002694	regulation of leukocyte activation	138/2368	427/14213	5.57370589681043e-16	2.52395982027232e-13	
BP	GO:0048872	homeostasis of number of cells	102/2368	281/14213	8.49812177376681e-16	3.55221490143453e-13	
BP	GO:0002250	adaptive immune response	112/2368	325/14213	2.33886514674245e-15	9.07813800528462e-13	
BP	GO:0009617	response to bacterium	127/2368	389/14213	3.56157539036937e-15	1.29024004475114e-12	
BP	GO:0031349	positive regulation of defense response	98/2368	275/14213	1.22081242094754e-14	4.14618418464308e-12	
BP	GO:0001819	positive regulation of cytokine production	117/2368	354/14213	1.68597407840542e-14	5.38916655415004e-12	
BP	GO:0002237	response to molecule of bacterial origin	93/2368	258/14213	2.63519795489985e-14	7.95536982606989e-12	
BP	GO:0097529	myeloid leukocyte migration	65/2368	155/14213	7.01441789008481e-14	2.00612351656425e-11	
BP	GO:0002262	myeloid cell homeostasis	66/2368	160/14213	1.15707260927156e-13	3.14376627939082e-11	
BP	GO:0030100	regulation of endocytosis	80/2368	213/14213	1.36568940287795e-13	3.53388391201848e-11	
BP	GO:0032943	mononuclear cell proliferation	88/2368	246/14213	2.17033695549989e-13	5.36073228008472e-11	
BP	GO:0030335	positive regulation of cell migration	135/2368	444/14213	2.32113409454902e-13	5.48393159555626e-11	
BP	GO:0042110	T cell activation	127/2368	410/14213	2.79118433990046e-13	6.29549704287216e-11	
BP	GO:2000147	positive regulation of cell motility	137/2368	454/14213	2.89634571350394e-13	6.29549704287216e-11	
BP	GO:0002685	regulation of leukocyte migration	66/2368	163/14213	3.29027733497889e-13	6.87667963010588e-11	
BP	GO:0030595	leukocyte chemotaxis	67/2368	167/14213	3.65987288004733e-13	7.13073770734008e-11	
BP	GO:0046651	lymphocyte proliferation	87/2368	244/14213	3.67428516388521e-13	7.13073770734008e-11	
BP	GO:0006909	phagocytosis	65/2368	160/14213	4.14212283552649e-13	7.76148120284515e-11	

Appendix Table S1. The most significantly enriched GO terms sorted by the *padj* value.
