# Supplementary material for: CD47-amyloid-β-CD74 signaling triggers adaptive immunosuppression in sepsis
Source: EMBO Rep. 2025 Apr 4;26(10):2683–714. doi: 10.1038/s44319-025-00442-4 (PMC12116991; doi:10.1038/s44319-025-00442-4)
Supplement: Supplementary file 6 — Source data Fig. 6 [file 44319_2025_442_MOESM6_ESM.zip › Source data Figure 6/Figure 6B.docx]

**Source Figure 6B**

**WT-Sham-24h WT-Mild-24h WT-CLP-24h**


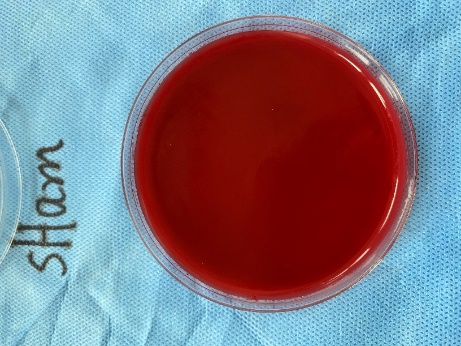

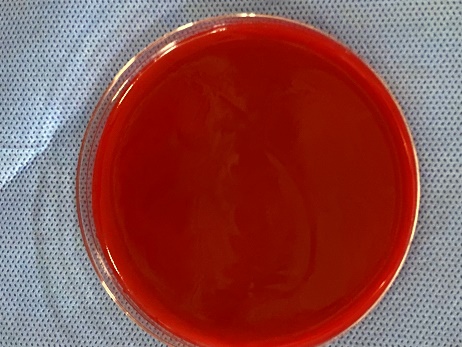

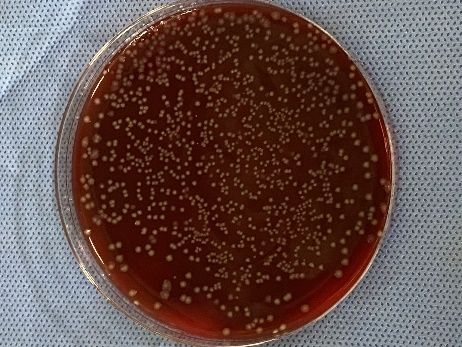


**WT-CLP-Anti-CD47-24h CD47-KO-CLP-24h**


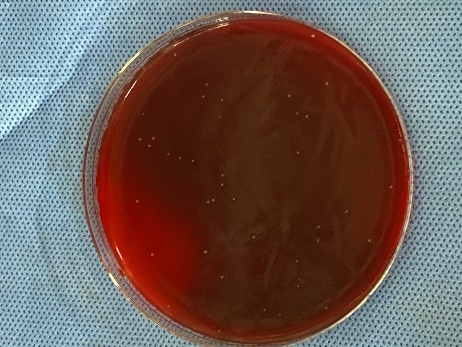

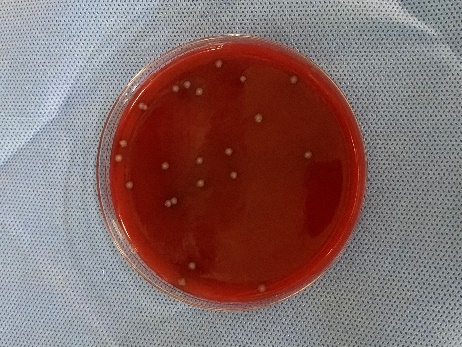


**WT-Sham-48h WT-Mild-48h WT-CLP-48h**


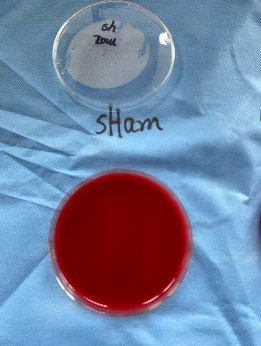

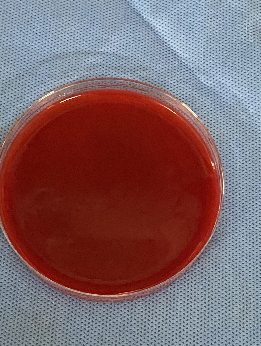

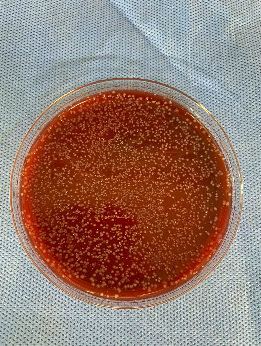


**WT-CLP-Anti-CD47-48h CD47-KO-CLP-48h**


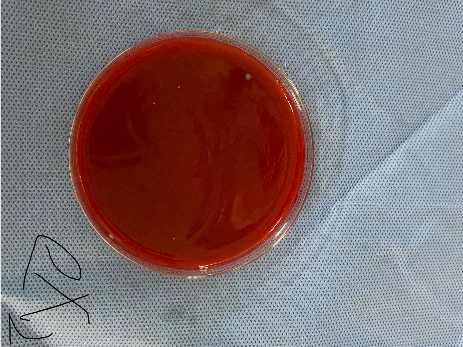

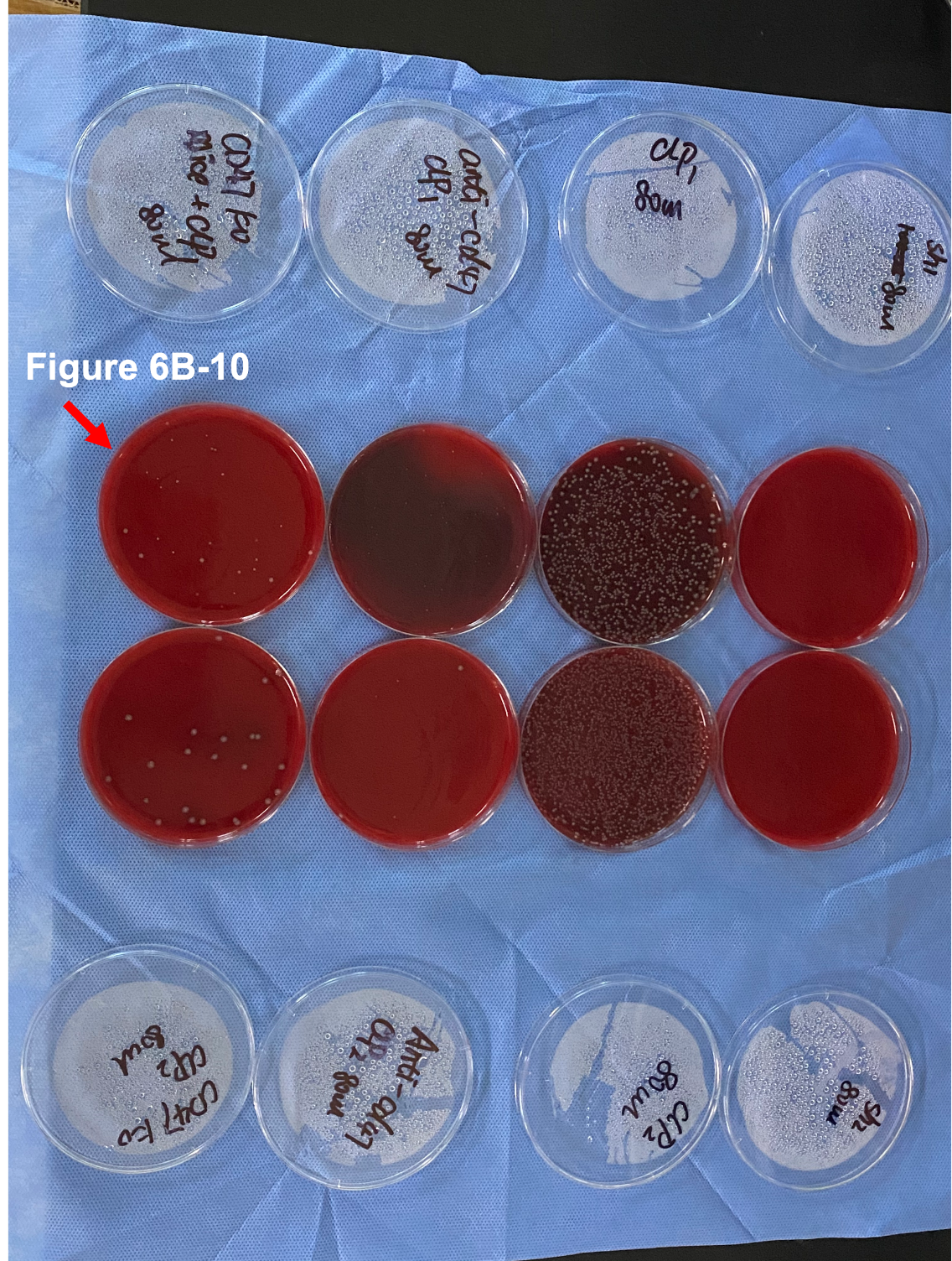


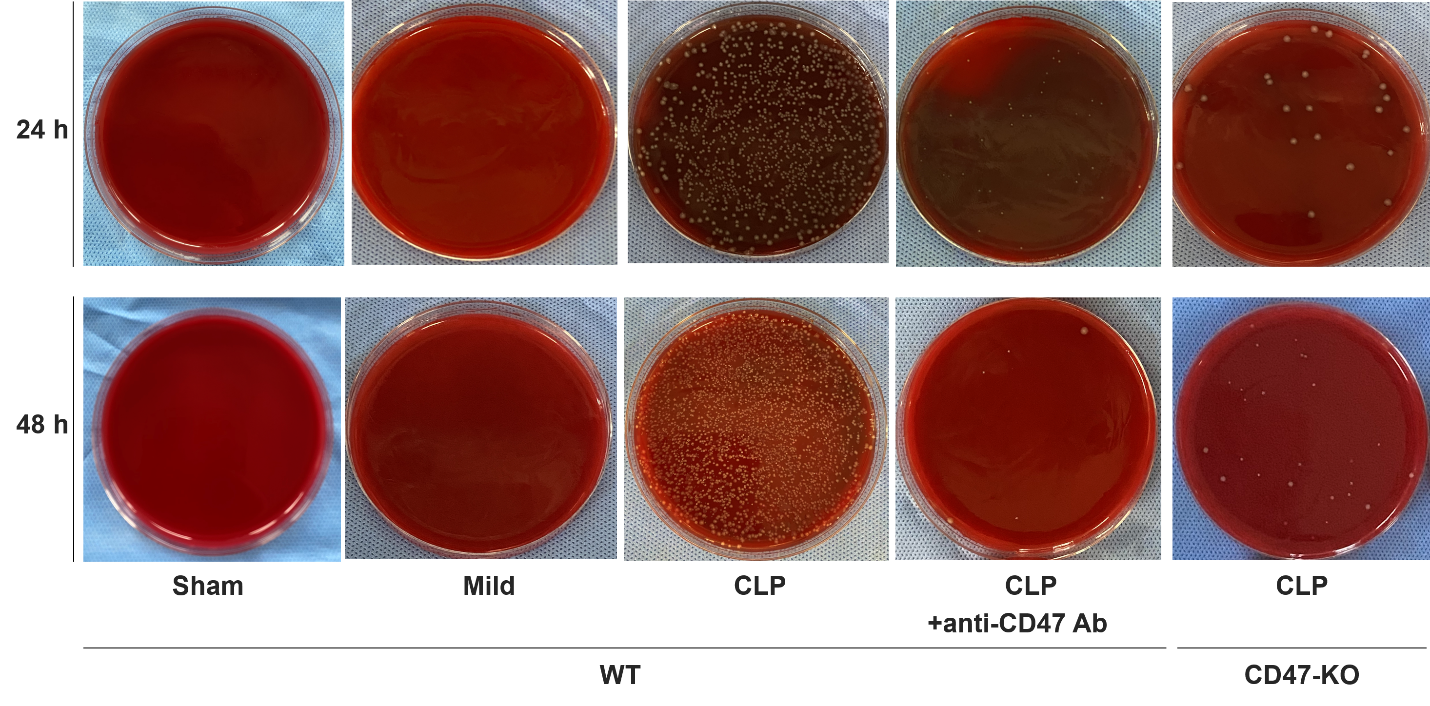


**
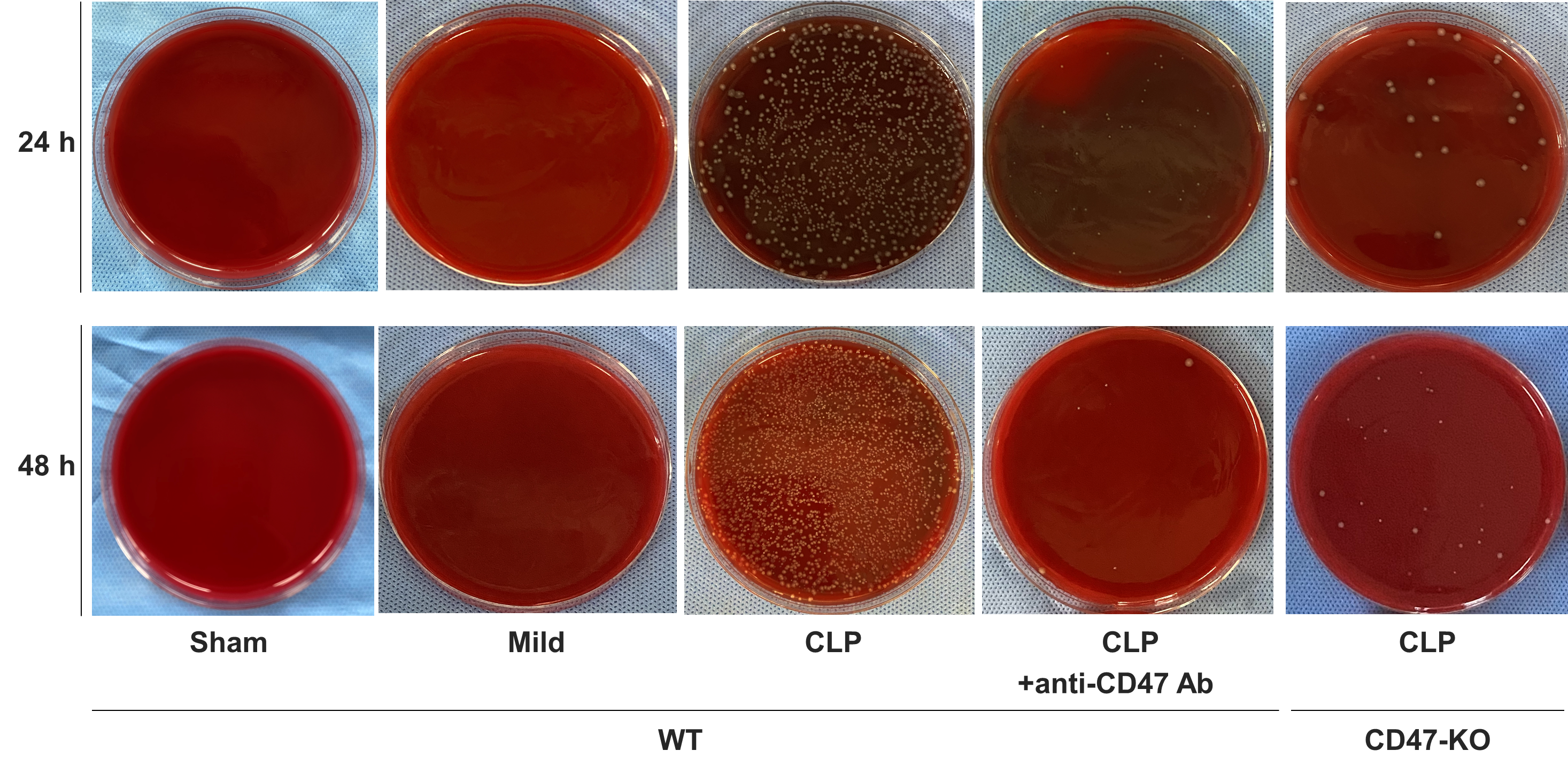
**
