# Supplementary material for: CD47-amyloid-β-CD74 signaling triggers adaptive immunosuppression in sepsis
Source: EMBO Rep. 2025 Apr 4;26(10):2683–714. doi: 10.1038/s44319-025-00442-4 (PMC12116991; doi:10.1038/s44319-025-00442-4)
Supplement: Supplementary file 7 — Source data Fig. 7 [file 44319_2025_442_MOESM7_ESM.zip › Source data Figure 7/Figure 7B.docx]

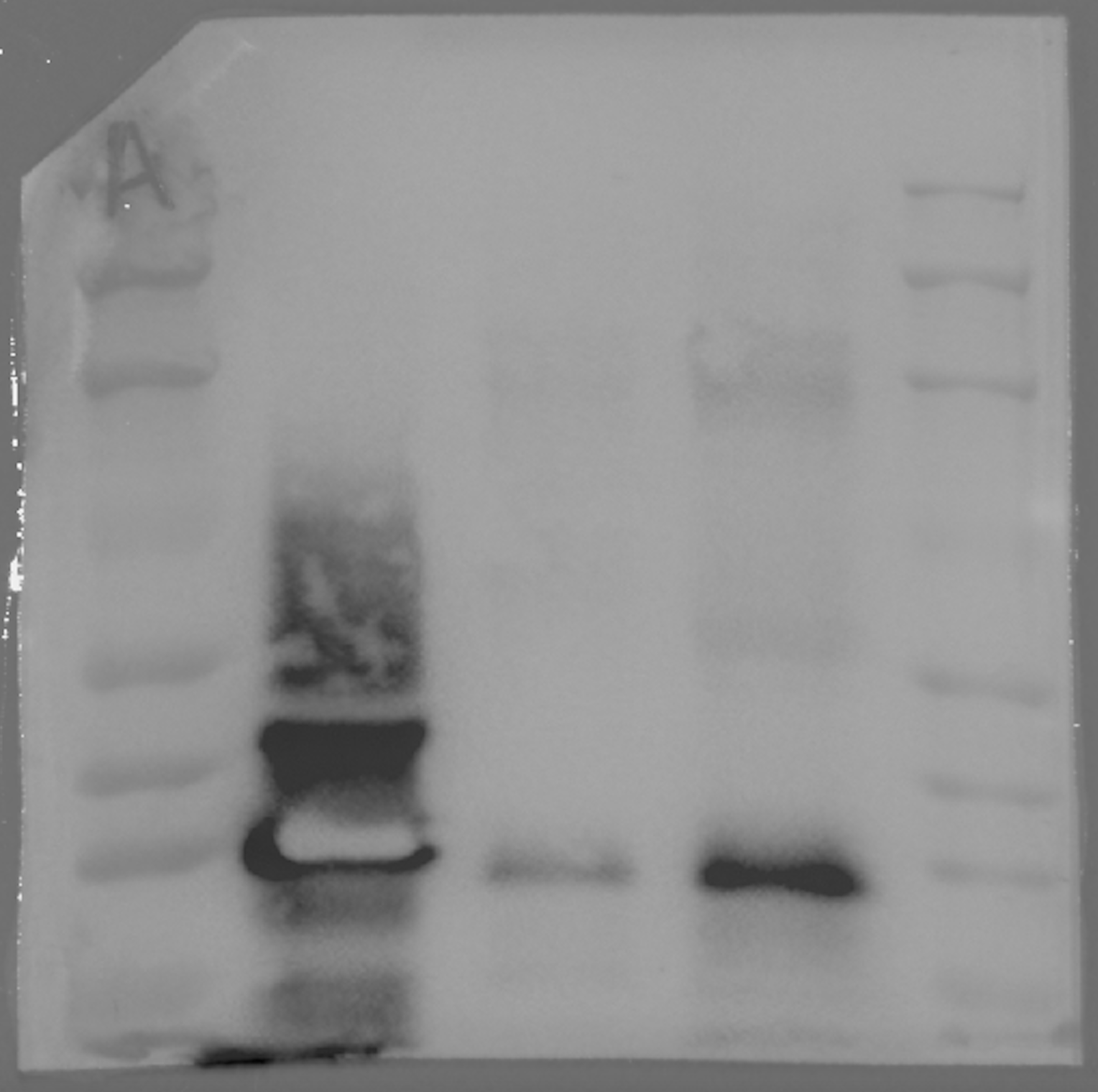


IB:CD74（41KDa/31KDa）

**30 kDa**

**40 kDa**

**50 kDa**

**70 kDa**

**100 kDa**

**150 kDa**

**250 kDa**

**KDa**

**Input**

**IP:**

**Anti-APP**

**IP:**

**IgG**

**Marker**

**WB:**

**Anti-CD74**

**(30 kDa)**

**Source Figure 7B**
