# Supplementary material for: CD47-amyloid-β-CD74 signaling triggers adaptive immunosuppression in sepsis
Source: EMBO Rep. 2025 Apr 4;26(10):2683–714. doi: 10.1038/s44319-025-00442-4 (PMC12116991; doi:10.1038/s44319-025-00442-4)
Supplement: Supplementary file 9 — Expanded View Figures [file 44319_2025_442_MOESM9_ESM.pdf]

Expanded View Figures

Figure EV1. Whole-mount scans of H&E-stained sections.

(**A**) Lung, (**B**) liver, and (**C**) kidney tissue sections from mice that underwent sham surgery, mild CLP surgery, and critical CLP surgery. Scale bars: full-slice scan, 1000 µm; first-level magnification field of view, 100 µm; second-level magnification field of view, 20 µm.

© The Author(s)

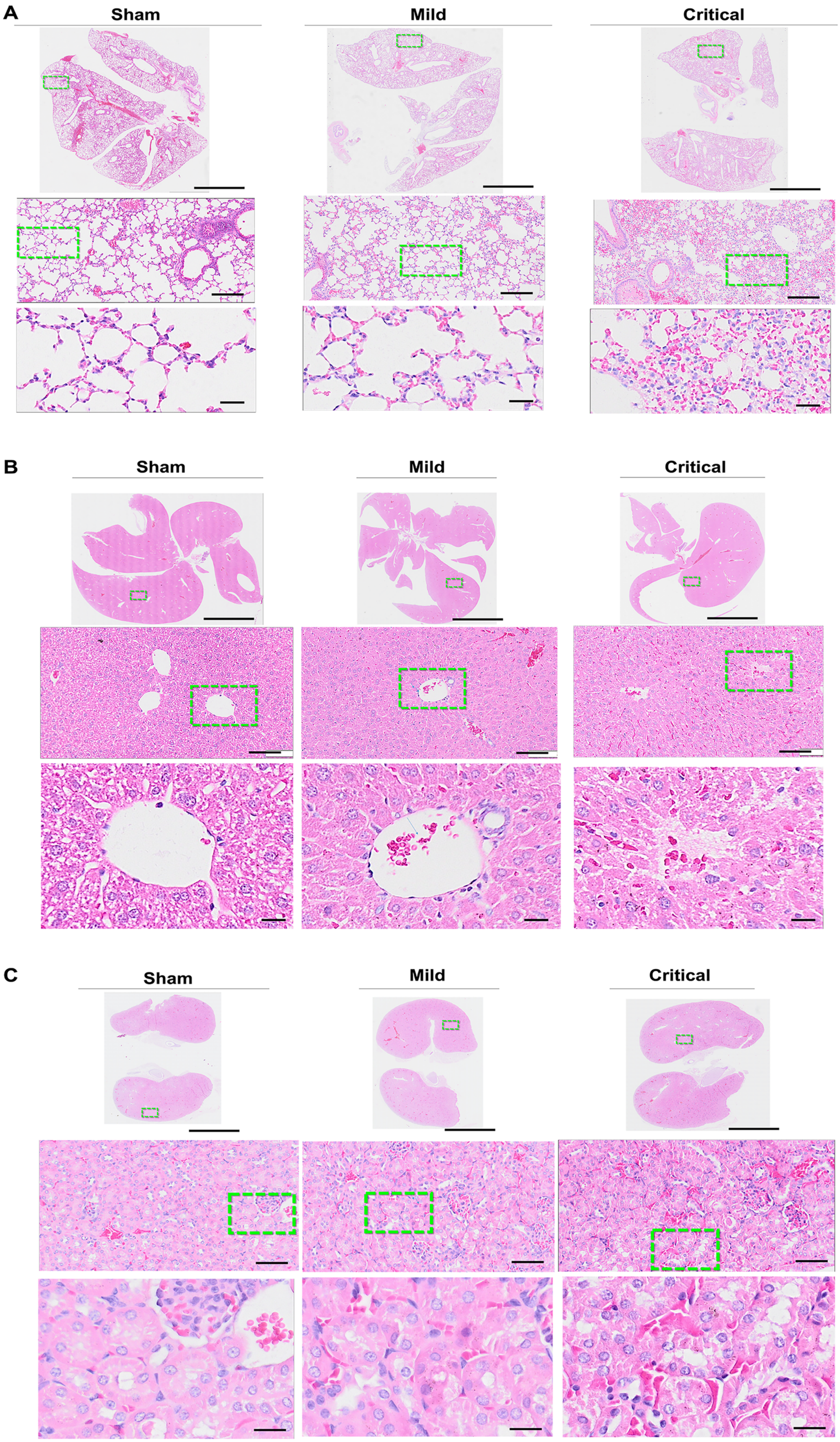
A
Sham
Mild
Critical
B
Sham
Mild
Critical
C
Sham
Mild
Critical

© The Author(s)

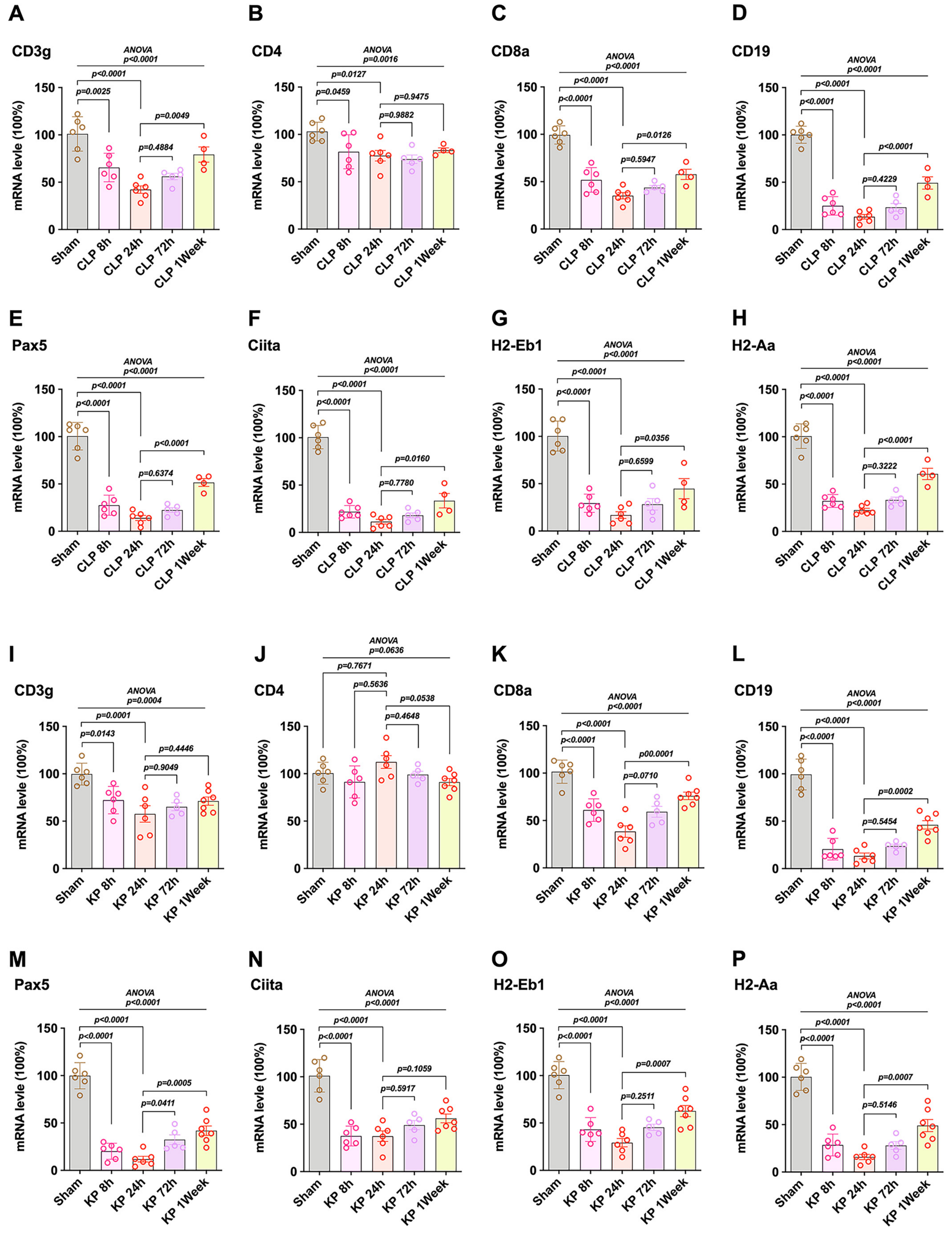
A
CD3g
ANOVA p<0.0001
p<0.0001
p=0.0025
p=0.0049
p=0.4884
mRNA levle (100%)
150
100
50
0
Sham
CLP 8h
CLP 24h
CLP 72h
CLP 1Week
B
CD4
ANOVA p=0.0016
p=0.0127
p=0.0459
p=0.9475
p=0.9882
mRNA levle (100%)
Sham
CLP 8h
CLP 24h
CLP 72h
CLP 1Week
C
CD8a
ANOVA p<0.0001
p<0.0001
p<0.0001
p=0.0126
p=0.5947
mRNA levle (100%)
Sham
CLP 8h
CLP 24h
CLP 72h
CLP 1Week
D
CD19
ANOVA p<0.0001
p<0.0001
p<0.0001
p<0.0001
p=0.4229
mRNA levle (100%)
Sham
CLP 8h
CLP 24h
CLP 72h
CLP 1Week
E
Pax5
ANOVA p<0.0001
p<0.0001
p<0.0001
p<0.0001
p=0.6374
mRNA levle (100%)
Sham
CLP 8h
CLP 24h
CLP 72h
CLP 1Week
F
Ciita
ANOVA p<0.0001
p<0.0001
p<0.0001
p=0.0160
p=0.7780
mRNA levle (100%)
Sham
CLP 8h
CLP 24h
CLP 72h
CLP 1Week
G
H2-Eb1
ANOVA p<0.0001
p<0.0001
p<0.0001
p=0.0356
p=0.6599
mRNA levle (100%)
Sham
CLP 8h
CLP 24h
CLP 72h
CLP 1Week
H
H2-Aa
ANOVA p<0.0001
p<0.0001
p<0.0001
p<0.0001
p=0.3222
mRNA levle (100%)
Sham
CLP 8h
CLP 24h
CLP 72h
CLP 1Week
I
CD3g
ANOVA p=0.0004
p=0.0001
p=0.0143
p=0.4446
p=0.9049
mRNA levle (100%)
Sham
KP 8h
KP 24h
KP 72h
KP 1Week
J
CD4
ANOVA p=0.0636
p=0.7671
p=0.5636
p=0.0538
p=0.4648
mRNA levle (100%)
Sham
KP 8h
KP 24h
KP 72h
KP 1Week
K
CD8a
ANOVA p<0.0001
p<0.0001
p<0.0001
p00.0001
p=0.0710
mRNA levle (100%)
Sham
KP 8h
KP 24h
KP 72h
KP 1Week
L
CD19
ANOVA p<0.0001
p<0.0001
p<0.0001
p=0.0002
p=0.5454
mRNA levle (100%)
Sham
KP 8h
KP 24h
KP 72h
KP 1Week
M
Pax5
ANOVA p<0.0001
p<0.0001
p<0.0001
p=0.0005
p=0.0411
mRNA levle (100%)
Sham
KP 8h
KP 24h
KP 72h
KP 1Week
N
Ciita
ANOVA p<0.0001
p<0.0001
p<0.0001
p=0.1059
p=0.5917
mRNA levle (100%)
Sham
KP 8h
KP 24h
KP 72h
KP 1Week
O
H2-Eb1
ANOVA p<0.0001
p<0.0001
p<0.0001
p=0.0007
p=0.2511
mRNA levle (100%)
Sham
KP 8h
KP 24h
KP 72h
KP 1Week
P
H2-Aa
ANOVA p<0.0001
p<0.0001
p<0.0001
p=0.0007
p=0.5146
mRNA levle (100%)
Sham
KP 8h
KP 24h
KP 72h
KP 1Week

 © The Author(s)

Figure EV2. Expression of adaptive immune-related genes over time during sepsis.

Real-time RT-PCR was used to detect the mRNA levels of adaptive immune-related genes at 8 h to 1 week after CLP surgery (**A–H**) and *K.p.* infusion (**I–P**). Data are shown as mean ± SD. The number of mice per group (n-number, biological replicates) was indicated by the points in the scatter plot. Significance was assessed by one-way ANOVA followed by Tukey's multiple comparison test. The exact *P* values are shown above the bars.

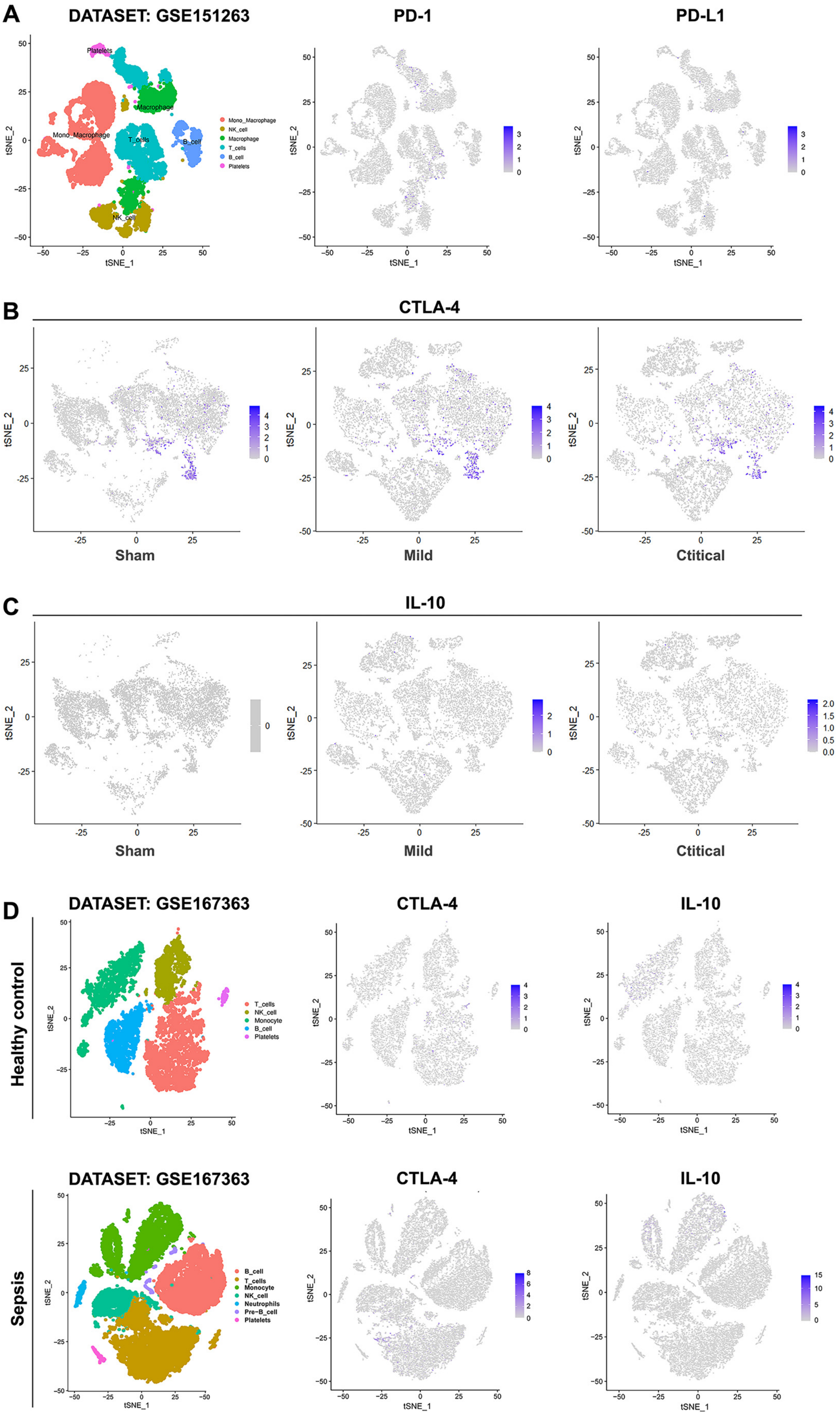
A
DATASET: GSE151263
PD-1
PD-L1
Mono_Macrophage
NK_cell
Macrophage
T_cells
B_cell
Platelets
tSNE_1
tSNE_2
B
CTLA-4
Sham
Mild
Ctitical
C
IL-10
Sham
Mild
Ctitical
D
DATASET: GSE167363
CTLA-4
IL-10
Healthy control
T_cells
NK_cell
Monocyte
B_cell
Platelets
DATASET: GSE167363
CTLA-4
IL-10
Sepsis
B_cell
T_cells
Monocyte
NK_cell
Neutrophils
Pre−B_cell
Platelets

 © The Author(s)

Figure EV3. The expression level of canonical immunosuppression genes.

(**A**) The scRNA-Seq public dataset GSE151263 shows the expression pattern of PD-1 and PD-L1 in human PBMCs. (**B**, **C**) The scRNA-Seq data shows the expression of CTLA-4 and IL-10 in mice received sham, mild or critical CLP surgery. (**D**) The scRNA-Seq public dataset GSE167363 shows the expression pattern of CTLA-4 and IL-10 in human PBMCs.

© The Author(s)

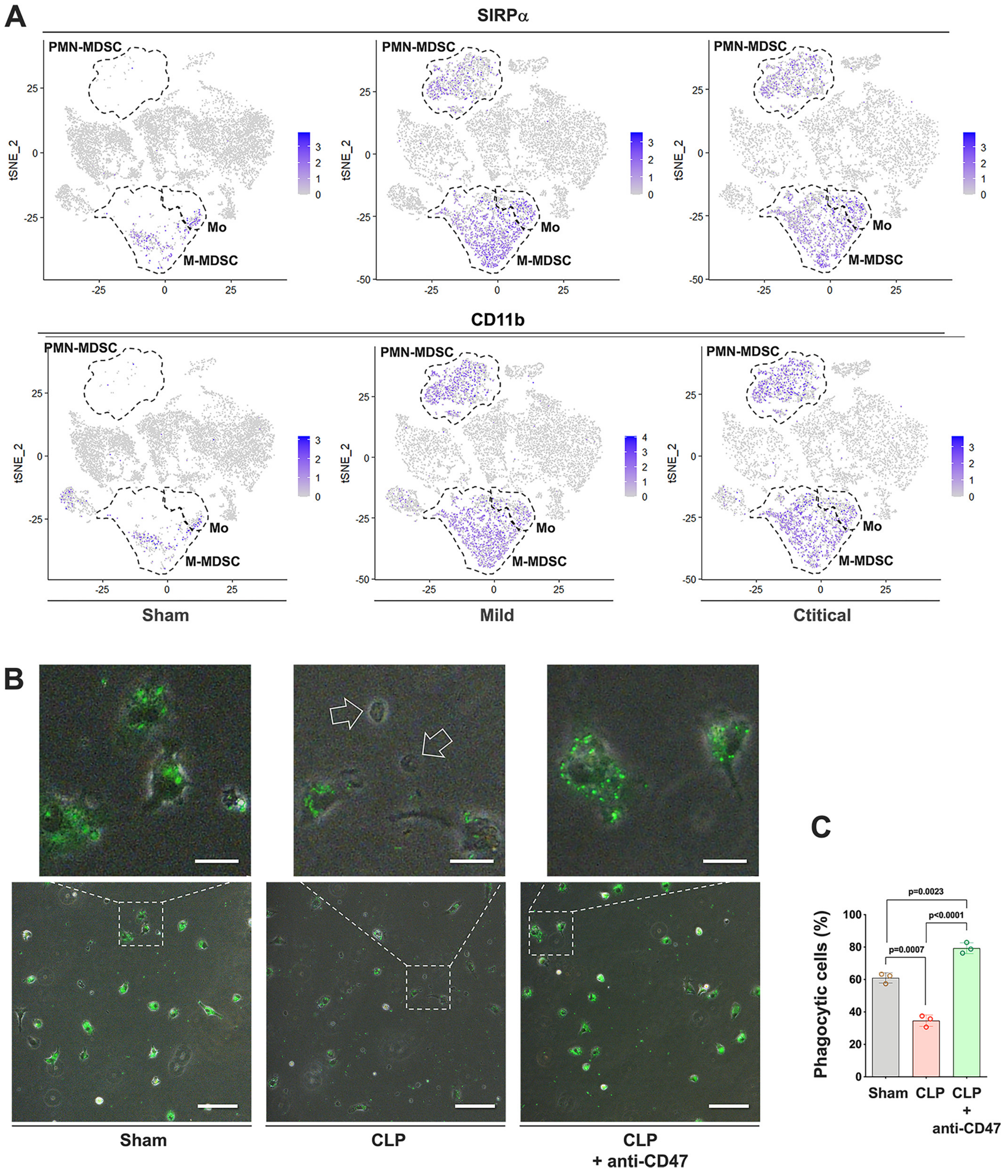
A
SIRPα
CD11b
PMN-MDSC
Mo
M-MDSC
tSNE_2
Sham
Mild
Ctitical
B
Sham
CLP
CLP
+ anti-CD47
C
Phagocytic cells (%)
p=0.0023
p<0.0001
p=0.0007
Sham
CLP
CLP
+
anti-CD47

 © The Author(s)

Figure EV4. CD47-SIRPα signaling reduces the phagocytotic ability of myeloid cells.

(**A**) Representative images showing the process of *E. coli* phagocytosis by myeloid cells from healthy control mice, CLP mice, or CLP mice treated with anti-CD47. (**B**) Phagocytosis rate (%) = (phagocytosing cells/all cells in a 60 × 60 mm dish) × 100%; each plot represents the Phagocytosis rate (%) from one 60 × 60 mm dish. Scale bars: upper field of view, 20 μm; lower field of view, 100 μm. (**C**) Data are shown as mean ± SD ($n = 3$, biological replicates) and were assessed by the 2-tailed parametric Welch's t test. The exact P values are shown above the bars.

© The Author(s)

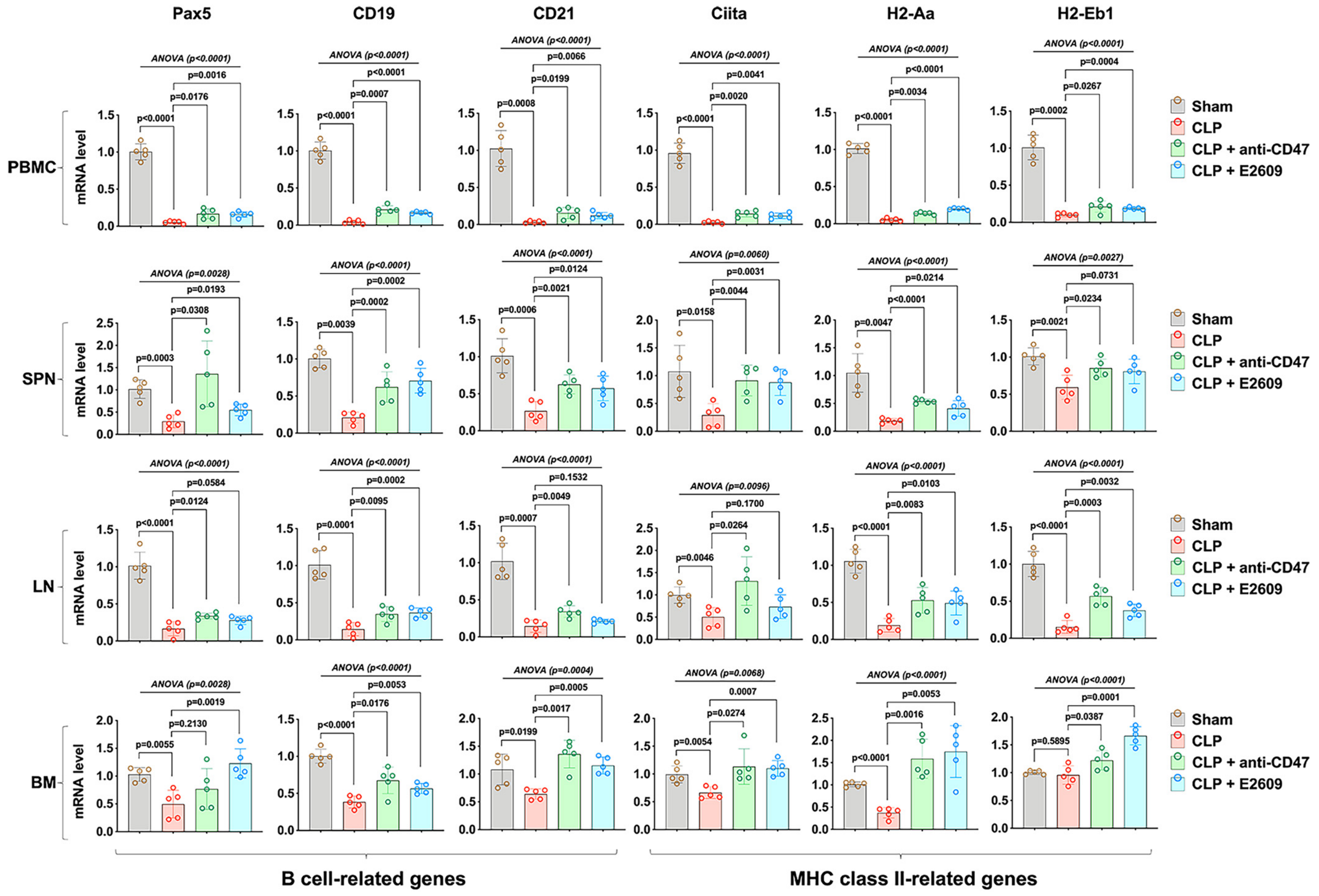

Figure EV5. Blocking CD47-amyloid-β signaling restores the transcription of genes that had been strongly suppressed in immune compartments.

The mRNA levels of Pax5, CD19, CD21, Ciita, H2-Aa, H2-Eb1 in PBMC, lymph nodes (LN), spleen (SPN), and bone marrow (BM) from control mice, CLP mice or CLP mice treated with anti-CD47 or E2609. The anti-CD47 antibody (2.5 mg/kg, i.v.) or Aβ inhibitor E2609 (6.5 mg/kg, i.p.) was injected 1 h after CLP surgery, and samples were collected 8 h after CLP. Data are shown as mean ± SD ($n = 5$, biological replicates) and were assessed by one-way ANOVA test followed by Tukey's multiple comparison. The exact *P* values are shown above the bars.

 © The Author(s)
